# Multidimensional Perspective on the Quality of Green Onion: Between the Visible, the Measurable, and the Perceived

**DOI:** 10.1002/fsn3.71565

**Published:** 2026-02-19

**Authors:** Leslie Walessa Castaño‐Tarazona, Juan Camilo Henao‐Rojas, Joaquín Guillermo Ramírez‐Gil

**Affiliations:** ^1^ Laboratorio de Agrocomputación y Análisis Epidemiológico, Departamento de Agronomía, Facultad de Ciencias Agrarias Universidad Nacional de Colombia Bogotá Colombia; ^2^ Corporación Colombiana de Investigación Agropecuaria (Agrosavia), Centro de Investigación La Selva Rionegro Colombia; ^3^ Grupo de Investigación en Sustancias Bioactivas, Facultad de Ciencias Farmacéuticas y Alimentarias Universidad de Antioquia Medellín Colombia

**Keywords:** *Allium fistulosum*, biofunctional compounds, consumer perception, data analytics, natural language processing, physicochemical parameters

## Abstract

Green onion ( *Allium fistulosum* ) is among the most highly consumed vegetables in the Andean region and parts around the world, playing a central role in food security, rural livelihoods, and local culinary identity. Green onion lacks a clear and standardized definition of quality, limiting commercial differentiation and value generation in national and international markets. Existing approaches remain fragmented, focusing on isolated attributes without integrating cultural, perceptual, origin‐related, and biofunctional dimensions into a unified framework. This study introduces an interdisciplinary methodological framework structured in five phases: (1) bibliometric analysis, (2) meta‐analysis of physicochemical parameters and biofunctional compounds, (3) exploration of digital trends, (4) agronomic field observation, and (5) consumer perception analysis. The approach integrates direct and indirect information sources across the value chain and applies data analytics and natural language processing to reach a multidimensional definition of green onion quality. Results indicate that quality in green anion is determined by physical attributes such as firmness, color, pseudostem thickness, freshness, and the absence of visible damage, together with biofunctional components including flavonoids (38.4–73.3 mg quercetin/100 g) and sulfur compounds (71.6–353.6 μmol/g). Unlike earlier studies, our findings demonstrate that quality is a multidimensional and territorialized concept, shaped by cultivation practices, climatic conditions, and cultural contexts. According to consumer preferences, the most important attributes associated with the quality of green anion are freshness (93.6%), pseudostem thickness (89.3%), and absence of damage (87.1%). These results support the development of technical protocols that unify objective criteria with local knowledge, while proposing a replicable model for other traditional crops, with applications in quality standardization, sustainable production, and commercial valorization.

## Graphical abstract

This graphical abstract presents a five‐phase framework to define green onion quality by integrating literature evidence, physicochemical and bioactive data, digital trends, field damage observations, and consumer perception, providing a multidimensional and measurable quality assessment.

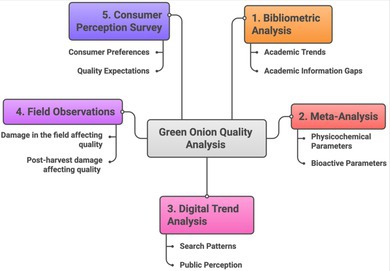

## 1 Introduction

The quality of horticultural products is a priority in agri‐food systems due to its central role in human nutrition and its direct impact on the competitiveness of national and international markets (Nicola and Fontana 2010; Zhao et al. 2024). The increasing demand for fresh, healthy, and high‐value foods has raised standards for production, handling, and marketing, particularly for perishable products such as vegetables, where quality evaluation requires an integrated assessment of visual, sensory, physical, chemical, nutritional, and organoleptic attributes (Fallik and Ilic 2018; Kyriacou and Rouphael 2018). Within this context, green onion ( *Allium fistulosum* L.) represents an essential crop in the global agri‐food system, influenced by evolving international trade, market globalization, consumer preference for natural products, and food waste reduction strategies (Kayat et al. 2021; Kim et al. 2003). In Colombia, green onion is one of the most economically and socially important vegetables, predominantly cultivated in cold‐climate regions such as the Cundiboyacense highlands, eastern Antioquia, and the Santanderes, where it plays a key role in regional production systems and food security (Bautista Peña 2020; Galeano Mendoza et al. 2018).

Due to its culinary versatility, high consumption frequency, and functional properties, the green onion has established itself as a food of scientific interest because of its phytochemical diversity and therapeutic potential (Balkrishna et al. 2023; Zhao et al. 2021). The *Allium* genus is characterized by a wide range of secondary metabolites with biological activity, such as sulfur compounds, flavonoids, saponins, phytosterols, and terpenes, compounds that have been extensively studied for their antioxidant, anti‐inflammatory, antimicrobial, antitumor, and cardioprotective effects (Wang, Zheng, et al. 2023; Yan et al. 2023). This bioactive profile contributes significantly to the nutritional and functional value of the genus, increasing its relevance in both applied research and food innovation (Kothari et al. 2020; Rocchetti et al. 2022).

Food quality has traditionally been defined as a set of physical, chemical, and sensory attributes that determine a food's acceptability to consumers or its commercial value in the market (Civille 1991; Mihafu et al. 2020) and, in the case of horticultural products, this definition has focused mainly on parameters such as size, color, firmness, moisture content, and the presence of defects, especially in fresh‐cut products (Liu et al. 1999; Toivonen and Brummell 2008). However, these approaches tend to favor a reductionist view, in which quality is evaluated in a fragmented manner, without considering the interactions between biological, technical, environmental, and, above all, social factors (Ibaraki et al. 1997; Wang, Qiao, et al. 2023).

For its part, a modern and holistic vision of quality in horticultural products could be defined as the inherent capacity of a product to satisfy both the requirements of the end consumer and the demands of the commercial chain, taking into account organoleptic, nutritional, physical, chemical, and microbiological attributes (Carmona Bayonas 2022; Gisbert Mullor 2023). This concept has evolved to integrate subjective and objective aspects of a product from the consumer's point of view, taking into account parameters such as safety, sensory qualities, origin, and the nature of the provider or seller, among others (Liverani et al. 2019; Ramírez‐Gil et al. 2019). This comprehensive approach, which has been termed “multidimensional quality”, allows for a more accurate assessment of product performance throughout the agri‐food chain, optimization of production and post‐harvest practices, and definition of objective criteria that respond to both market demands and consumer preferences (Emam 2009; Ramírez‐Gil et al. 2019). This is determined by factors such as the sanitary condition of the crop, climatic conditions, harvesting methods, and handling during storage, all of which have a direct impact on the final quality of the product (Choi et al. 2024; Medina‐Jaramillo et al. 2022).

In response to the growing complexity of food quality assessment, recent methodological advances have incorporated natural language processing (NLP) and data science to enable systematic and scalable analyses of large volumes of scientific information (Castaño‐Tarazona, Hernández‐Sánchez, et al. 2025; Ospina‐Sanchez et al. 2025). NLP facilitates the identification of research trends, knowledge gaps, and the automated extraction of physicochemical, bioactive, and sensory attributes from open‐access literature using programming environments and specialized libraries (Chergui and Kechadi 2022; Yang and Xu 2021). Complementarily, data science integrates statistical, multivariate, and machine learning techniques to analyze heterogeneous datasets, enhancing the accuracy, speed, and robustness of food quality evaluation while overcoming the limitations of traditional univariate approaches (Bautista‐Romero et al. 2025; Dhal and Kar 2025).

On similar lines, with advances in analytical methods, the use of open data and digital sources has enabled the incorporation of behavioral trends and public interest into agri‐food research through platforms such as Google Trends (Kaur 2024; Ospina‐Sanchez et al. 2025). These tools allow the examination of search patterns related to specific products at national and international scales, providing insights into consumer preferences, demand seasonality, and product perception across different cultural contexts (Mulder 2019; Schaub et al. 2020). Such analyses complement academic evidence by offering a dynamic and real‐time interpretation of public needs, perceptions, and uses of agricultural products (Kaur 2024; Thirunavukarasu 2021). At the same time, field‐based observational data in small‐scale agricultural systems such as records of visual crop damage, weather conditions, and producer assessments can be integrated into simple digital platforms and combined with IoT sensors to generate accessible, low‐cost quality indicators (Falcão et al. 2024). Systematic evidence further demonstrates that the integration of heterogeneous sensory, observational, and quantitative data through data mining and machine learning approaches enhances the development of adaptive, multidimensional frameworks for crop quality management (Chergui and Kechadi 2022), while participatory citizen science initiatives validate the reliability of aggregated farmer‐generated data for classifying varieties and maturity stages (Steinke et al. 2017).

In this regard, strategic analytical approaches that integrate bibliometric review, NLP, digital trend analysis, consumer perception, and direct field observation have emerged as effective methodological tools for understanding how knowledge related to agri‐food products is constructed across scientific, non‐scientific, and social domains (Castaño‐Tarazona, Hernández‐Sánchez, et al. 2025; Castaño‐Tarazona, Valbuena‐Gaona, and Ramírez‐Gil 2025). Recent studies on horticultural value chains demonstrate that holistic frameworks encompassing production, post‐harvest, and market stages enable the identification of key actors, critical activities, and their impacts on product performance throughout the value chain (Castaño‐Tarazona, Hernández‐Sánchez, et al. 2025; Castaño‐Tarazona, Valbuena‐Gaona, and Ramírez‐Gil 2025).

However, despite the socio‐economic importance of green onion, there is a clear knowledge gap regarding its multidimensional quality, together with a marked disconnection among value chain stakeholders concerning quality parameters and their interpretation. To address this limitation, the present study aims to unify a concept of multidimensional quality for green onion by introducing an interdisciplinary methodological framework structured in five sequential phases: (1) bibliometric analysis, (2) meta‐analysis of physicochemical parameters and biofunctional compounds, (3) exploration of digital trends, (4) agronomic field observation, and (5) consumer perception analysis. This integrated approach combines direct data sources, such as field observations and consumer surveys, with indirect sources including scientific literature, digital trends, and open data, to establish a robust baseline for the multidimensional quality of green onion across the value chain.

## 2 Materials and Methods

### 2.1 Basic Description of the Proposed Methodological Approach

**FIGURE 1:**
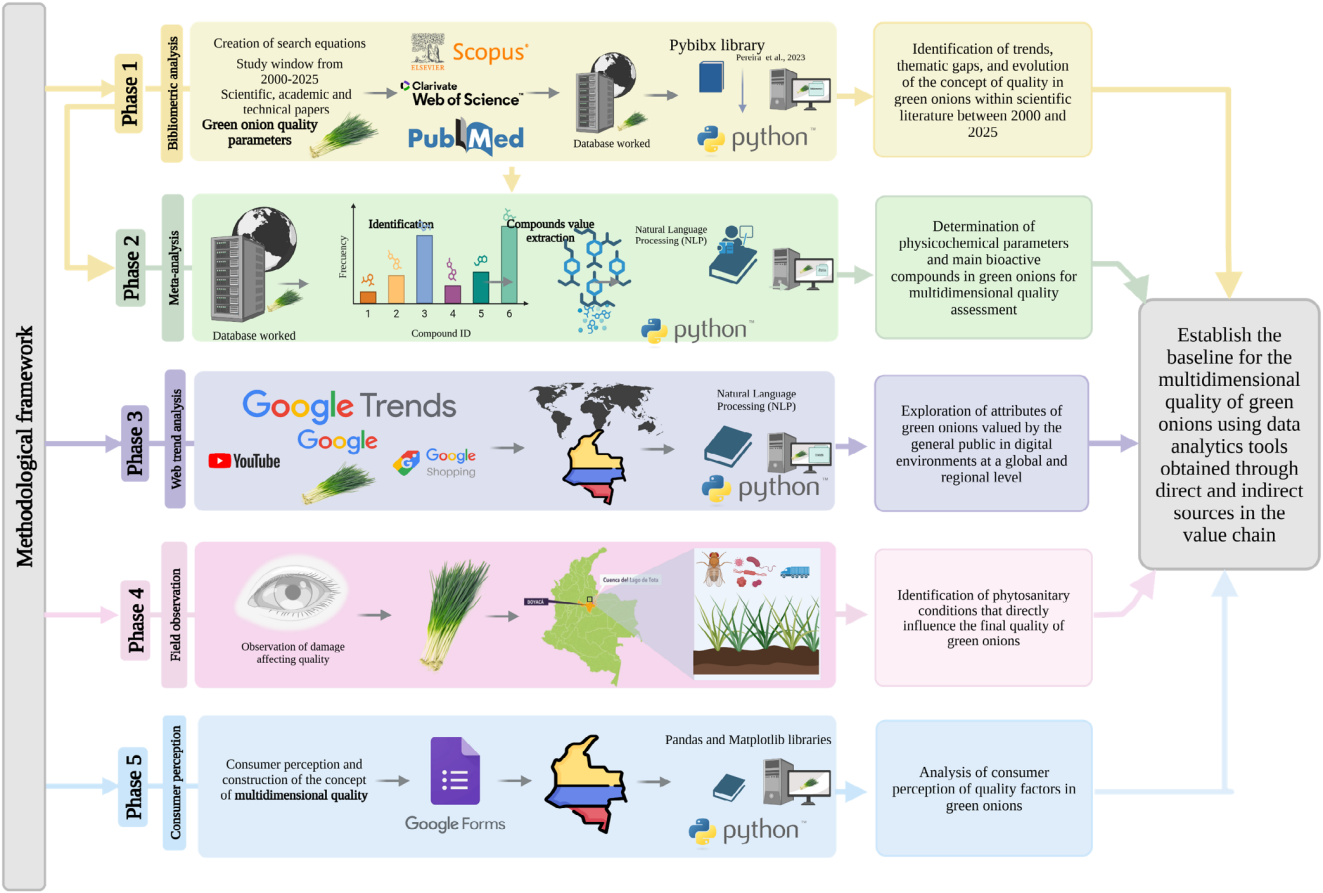
Outline of the methodological approach used to develop this study, structured in five sequential stages. The framework integrates bibliometric analysis, meta‐analysis of physicochemical and bioactive parameters, digital trend analysis, field observation, and consumer perception to characterize the multidimensional quality of green onion across the value chain.

This study was developed using a composite methodology divided into five complementary phases designed to establish multidimensional quality parameters for green onion. The phases were organized sequentially and articulately, allowing the concept of quality to be addressed from five key perspectives: scientific, technical, digital, productive, and social, represented respectively in each phase (Figure 1). In our approach, the phases were presented sequentially; however, this does not imply that future analyses must replicate this order. The sequence was intentionally designed to move from tangible, scientifically validated components supported by published evidence and technical parameters through an intermediate digital trends and productive phase to less tangible elements such as consumer quality perception. This structure facilitates clarity and interpretability rather than imposing a rigid analytical pathway. Our work aims to establish a logical connection across value chain stages by integrating multidimensional quality concepts, ultimately supporting the development of a comprehensive and adaptable quality framework for green onion production.

In basic descriptive terms, the first phase consisted of a bibliometric analysis aimed at identifying trends, information gaps, and thematic developments regarding green onion and their post‐harvest treatment. Hyperparameters such as the analysis period, language, document type, and keywords used were employed, with the data being processed using Python libraries and scientific visualization tools. In the second phase, a NPL‐assisted data extraction was carried out to extract quantifiable physicochemical and bioactive parameters associated with quality, using NLP techniques and manual review of specialized literature.

The third phase addressed the analysis of digital trends using platforms such as Google Trends, YouTube, and Google Shopping, with the aim of identifying search patterns and public perception related to green onion. In the fourth phase, field observations were carried out in the municipalities of Boyacá and Antioquia to record agronomic conditions, phytosanitary problems, and post‐harvest management practices, including the identification of damage affecting visual quality. Finally, in the fifth phase, a survey was conducted among consumers of the product to ascertain their perception of product quality, using a Google Forms questionnaire with structured open‐ended questions and Likert scales. Together, the activities in each phase allowed us to address the main objective, which was to establish a baseline for green onion quality using analytical tools and direct and indirect data in the value chain (Figure 1).

### 2.2 Phase 1: Bibliometric and Scientometric Analysis

This phase acts as a cross‐cutting level of technical and conceptual knowledge, allowing us to identify how the quality of green onion has been understood, evaluated, and assessed at different stages, especially in the research segments. The objective was to explore and analyze the available scientific landscape on green onion, with an emphasis on the factors that influence its post‐harvest quality. To this end, a bibliometric and scientometric analysis was carried out based on indexed publications from scientific and academic databases. This phase was integrated as a starting point to identify the evolution of scientific interest in the product, the topics that have been addressed, the countries and authors leading the research, and the gaps in knowledge.

For this, three recognized databases were used: Scopus, Web of Science, and PubMed, ensuring broad international coverage across regions, disciplines, and publication traditions. Each has different technical characteristics, so it was necessary to adapt the same search strategy to their respective syntaxes, maintaining consistent selection criteria. The search equations focused on identifying publications related to green onions and their post‐harvest characteristics, encompassing three aspects: (1) post‐harvest, organoleptic, and sensory quality, (2) physicochemical characteristics and bioactive compounds, and (3) trends and perceptions. In the equation, each word was entered in English and Spanish, and the search range was set between 2000 and 2025. No geographical restrictions were applied during the bibliometric search, allowing the inclusion of scientific literature from Asia, Europe, North America, Africa, and Latin America. This global coverage enabled the identification of international research trends and dominant thematic clusters beyond local or regional contexts. These results provide key technical and conceptual support for constructing a coherent, well‐founded, and contextualized multidimensional definition of quality:

(“quality” OR “postharvest quality” OR “sensory quality” OR “organoleptic quality” OR “fresh quality” OR “quality parameters” OR “calidad” OR “calidad poscosecha” OR “calidad sensorial” OR “calidad organoléptica” OR “parámetros de calidad” OR “physicochemical properties” OR “chemical composition” OR “nutritional content” OR “volatile compounds” OR “bioactive compounds” OR “propiedades fisicoquímicas” OR “composición química” OR “contenido nutricional” OR “compuestos volátiles” OR “compuestos bioactivos” OR “trends” OR “perceptions” OR “factors” OR “variables” OR “tendencias” OR “percepciones” OR “factores” OR “variables”) AND (“cebolla de rama” OR “Allium fistulosum” OR “green onion” OR “welsh onion” OR “spring onion” OR “cebolla larga”) AND (publication date between 2000 and 2025).

The bibliometric analysis was performed on a total of 250 documents, which were classified as 208 scientific articles, 2 books, 4 book chapters, 27 conference papers, 1 manuscript, and 8 literature reviews. The documents were downloaded from each database following their export protocols: BibTex, csv, or txt. Subsequently, they were processed in the Google Colab environment using the open‐source programming language Python. Data analysis was performed using the specialized Python library pybibx, designed to work with files exported from the Scopus, Web of Science, and PubMed databases. This tool integrates artificial intelligence capabilities and advanced textual analysis, allowing for a comprehensive approach to academic data (Pereira et al. 2023).

Subsequently, duplicate documents were cleaned, classified by document type, and evaluated for quality using a file quality report. An exploratory data analysis (EDA) was then performed, providing detailed information on the number of publications, authors, institutions, countries, languages, publication sources, citations, keywords, and other key indicators. Multiple interactive visualizations were also generated. These included word clouds and bar charts based on titles, abstracts, and keywords; maps showing the evolution of keywords, authors, and sources over time, productivity charts by country, citation trajectories, and publication year spectroscopy (RPYS).

A co‐occurrence analysis of keywords in the abstracts was performed based on the documents collected. To do this, VOSviewer software was used, a computer tool specialized in the construction and visualization of bibliometric maps, which allows the structure and evolution of scientific knowledge in a given area to be understood (Kumar et al. 2023; Wong 2018). These procedures made it possible to visualize patterns, identify influential authors, detect thematic clusters, and evaluate the evolution of scientific knowledge regarding the multidimensional quality of green onion in the post‐harvest period. All processing and visualization were performed using reproducible code, ensuring the traceability of results and the possibility of replication.

This phase established the current global state of the scientific literature on green onion, its post‐harvest management, and quality‐related research, providing the conceptual and technical foundation for the subsequent identification and extraction of specific quality parameters addressed in Phase 2.

### 2.3 Phase 2: NLP‐Assisted Data Extraction of Green Onion Quality Parameters

This phase focuses on the NLP assisted extraction and synthesis of quantitative data reported in studies of phase 1, representing an approach in a broad, non‐statistical sense. The objective of this phase was to identify and systematize the quantifiable parameters associated with the quality of green onion, based on the detailed analysis of scientific publications. NLP tools were used to automatically detect, filter, and extract numerical values and measurement units related to physicochemical and biofunctional attributes, enabling the efficient handling of large volumes of unstructured textual information. Similar NLP and text mining based approaches have been successfully applied in agricultural research to transform heterogeneous scientific reporting into structured, comparable datasets, improving reproducibility and supporting data driven quality assessment across studies (Ospina‐Sanchez et al. 2025).

For this, first search engines configured with artificial intelligence were used through the AI *Future House* platform (Skarlinski et al. 2025). This tool enabled the automated filtering of documents that mention measurable post‐harvest parameters, such as product size, shelf life, and other physical properties associated with preservation and handling. The filtering was based on NLP techniques focused on recognizing patterns of units of measurement. As a result, six key documents were selected, which were then analyzed manually to ensure the validity of the information extracted.

In parallel, an analysis was carried out to identify bioactive compounds present in the previously downloaded documents. The Python Counter library, belonging to the collections module, was used to count the frequency of mention of functional compounds, mainly sulfides and flavonoids, as these are the ones most frequently reported for Alliaceae (Beretta and Galmarini 2016). Subsequently, NLP‐assisted procedures were applied to abstracts using targeted keywords, and numerical values associated with these compounds were identified through unit‐based pattern recognition (e.g., mg/100 g) using regular expressions. To ensure data reliability, ambiguous matches and false positives were excluded through manual inspection, and all extracted values were verified to confirm their correspondence to the intended compound and measurement context.

The outcome of this phase was the systematization of physicochemical and biofunctional parameters reported in the literature, intended as a reference framework to guide future research and to support the interpretation of quality variations under field and post‐harvest conditions addressed in Phase 4.

### 2.4 Phase 3: Web Trend Analysis

The objective of this phase was to explore how green onion is perceived and related to in digital environments, especially in relation to attributes associated with its quality. This phase was integrated into the analysis to incorporate a perceptual dimension of the concept of quality, complementing the scientific evidence from phases 1 and 2. To do this, Google Trends was used, a free platform from Google Inc. that allows users to observe the frequency and evolution of searches for terms in different categories such as images, news, YouTube, Google Shopping, and the web. This tool makes it easy to identify temporal patterns, regions of greatest interest, and topics associated with the consumption of specific products (Jun et al. 2018; Mavragani et al. 2018).

Given that green onion is grown and consumed in specific regions of the world, search results directly related to its quality are still limited and do not provide sufficient comparative data on a global scale. For this reason, the information was extracted from related and representative terms such as “green onion” for international analysis, and “cebolla de rama” or “cebolla larga” for specific analysis of countries such as Colombia and other Spanish‐speaking contexts. This approach made it possible to identify temporal trends and search patterns associated with the consumption and perception of the product, including aspects such as culinary uses, freshness, preservation, health benefits, and subjective quality attributes.

The search was conducted on platforms such as Google, YouTube, and Google Shopping, which allowed us to identify how the product is interpreted and valued in different digital environments. The time frame considered was from January 1, 2008 to January 1, 2025, as this is the period in which stable and available information about the product can be found on digital platforms. The Google Trends location feature was used to identify the regions with the highest search volume, thus allowing for analysis of the geographical distribution of interest and possible differences in the perception of quality according to cultural and regional context.

For the processing and analysis of the extracted data, the collaborative environment Google Colab was used, employing the Python programming language. The NLTK (Natural Language Toolkit) and SpaCy libraries, specialized in NLP, were integrated in order to perform cleaning, segmentation, and semantic analysis of the texts related to the search terms. This methodology facilitated the identification of recurring themes, the evolution of associated terms, and the exploration of global and regional public interest in green onion.

This phase provided a perceptual and contextual layer to the concept of quality, allowing the prioritization of attributes identified in the literature according to public interest and use, and serving as a complementary guide for interpreting agronomic and post‐harvest observations.

### 2.5 Phase 4: Observation of Quality Parameters in the Field and Post‐Harvest in Agro‐Industry 0

Based on the quality parameters and contextual attributes identified in Phases 2 and 3, field and post‐harvest observations were conducted to examine how these physicochemical and functional traits are affected by biotic and abiotic factors under real production and handling conditions. The objective of this phase was to identify the causes of damage at the phytosanitary, physiological, mechanical, and post‐harvest handling levels that influence the final quality of green onions. This stage was integrated as a practical validation of the product under real conditions, and its inclusion allows the productive dimension to be incorporated into the concept of multidimensional quality. In particular, this was because it included the perception of damage and effect on quality directly by producers, and those who operate at agro‐industry level 0, who are paid directly by the marketer based on quality.

Two observations were made, the first in April 2025 in the Aquitania region of Boyacá (Colombia), an area known for its favorable agricultural conditions for growing green onion. Aquitania is characterized by a cold climate, suitable for the development of this type of crop, with altitudes ranging from 2600 to 3060 m above sea level, an average temperature of 11°C, and precipitation between 1500 and 2000 mm in a bimodal system (Cruz and Espinel 2017; Segura et al. 2015). A one‐hectare plot was selected, which was at harvest time. After the field observation, two warehouses for cleaning and packing the product were visited, both located in the urban center of the municipality, in order to observe quality parameters. The second observation was made in the municipality of Rionegro, Antioquia (Colombia), a town characterized by a temperate climate due to its altitude between 1800 and 2200 m above sea level, average temperatures between 18°C and 21°C, and annual rainfall of 1900 to 2500 mm (Ceballos‐Sierra and Dall'Erba 2021). Quality parameters were observed there on a 0.4‐ha plot.

To ensure the representativeness of each sample, an evaluation was carried out for each batch, integrating three types of sampling: random, stratified, and systematic grid sampling within the evaluated batch, which allowed the phytosanitary conditions of the crop to be recorded. The analysis focused on identifying damage associated with pests, diseases, physiological factors, and mechanical aspects present in the field. For each of these quality‐reducing factors, incidence values were calculated, understood as the percentage of affected plants relative to the total observed (*n* = 30).

### 2.6 Phase 5: Consumer Perception Analysis

The objective of this phase was to integrate consumer perception into the analysis, incorporating the social and subjective dimensions of quality. Unlike the previous phases, this sought to understand how the quality of green onion is valued and defined from the consumer's experience in the value chain. To this end, a survey was conducted using Google Forms, following the regulations for the collection, storage, processing, and use of data for academic purposes. The survey was divided into two sections. The first included questions regarding the place of purchase, product use, type of storage, post‐harvest duration, and the benefits of consumption. Overall, questions were implemented using the Likert scale format (Batterton and Hale 2017; Maldonado Luna 2012), created from a statement followed by a set of ordered response options, which reflected different levels of agreement or disagreement to assess the importance of key attributes associated with the quality of green onion and purchasing decisions. These attributes included aroma, physical appearance, price, texture, color, nutritional properties, firmness, flavor, size, and origin of the product. The second part of the survey related to questions about personal taste in certain quality parameters to discern preferences for attributes such as flavor, smell, and type of firmness, size, and color of the pseudostem and leaf of the green onion.

This form was distributed through social networks such as Facebook, WhatsApp, and LinkedIn, as well as corporate emails from the Universidad Nacional de Colombia and the Corporacion Colombiana de Investigacion Agropecuaria‐AGROSAVIA. Responses were obtained randomly, seeking the greatest possible representativeness in terms of age, gender, geographical location, socioeconomic status, among others. The analysis was based on a total of 115 responses, a sample size representative for consumer perception studies aimed at identifying quality evaluation criteria. The sample incorporated relevant structural diversity, including variation in geographic origin, age, gender, socioeconomic strata, and consumption preferences. In addition, the analytical methodology employed based on the integration of Likert scale analysis and NLP, an approach that prioritizes informational richness, semantic consistency and the recurrence of discursive patterns, focused on the consistency, recurrence, and semantic richness of the responses (Nadkarni et al. 2011). This type of approach has recently been supported in food science and consumer behavior studies (Castaño‐Tarazona, Hernández‐Sánchez, et al. 2025; Castaño‐Tarazona, Valbuena‐Gaona, and Ramírez‐Gil 2025).

Survey results were analyzed using descriptive statistics and expressed as relative frequencies and percentages, without performing population level statistical inference. Two tools were used to process the information in the collaborative environment Google Colab and R‐Studio, using Python and R as programming languages. The first was the Wordcloud library in Python, where PLN was used to analyze textual data and extract relevant information from each group of responses, allowing the creation of visualization graphs such as Wordclouds, which represented the frequency with which each word was repeated and related it to its size within the image. This allowed the identification of the most relevant terms and their subsequent analysis. The second tool was the Likert package in R‐Studio, which allowed the analysis of the level of importance of each attribute, being 1‐not important, 2‐not very important, 3‐moderately important, 4‐important, and 5‐very important, and their respective graphs. This package allows a numerical representation based on dispersion measurement for qualitative information.

## 3 Results

### 3.1 Overview of Scientific Research on Green Onion Through Bibliometric Analysis

**FIGURE 2:**
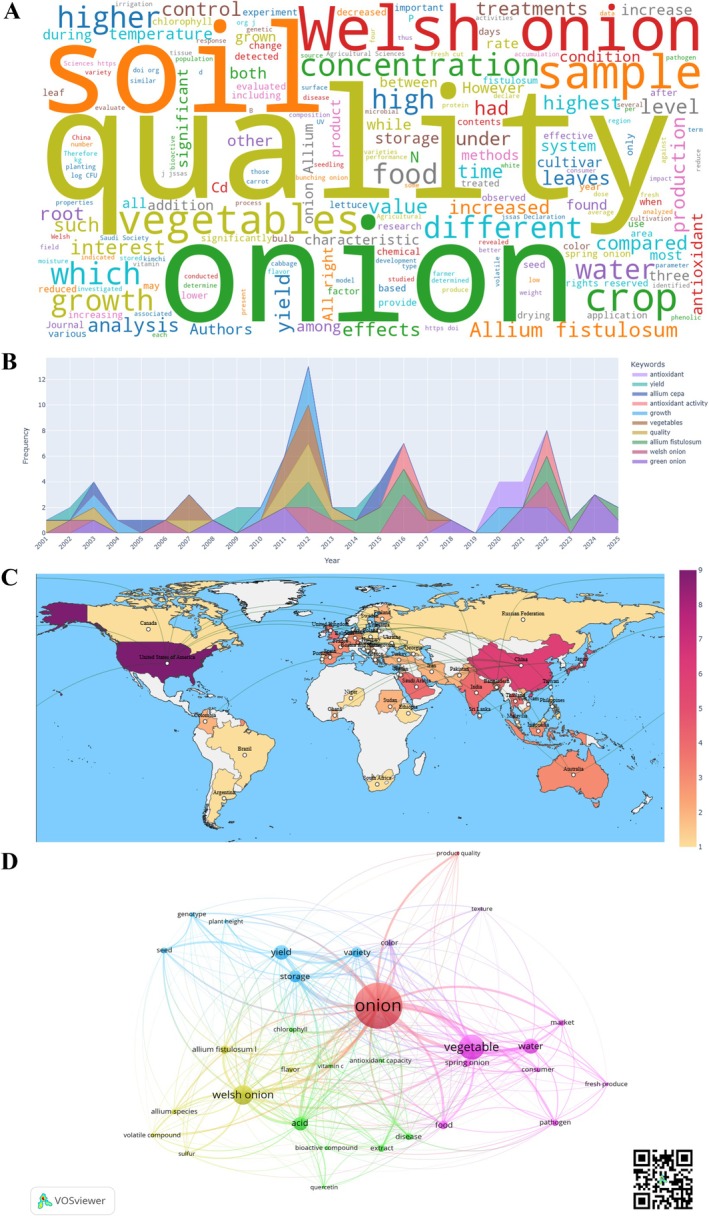
Bibliometric analysis of research on green onion ( *Allium fistulosum* ). (A) Word cloud generated from the frequency of terms in the abstracts of the documents analyzed, reflecting dominant research topics related to quality. (B) Temporal distribution of scientific production according to keyword frequency, illustrating changes in research focus over time. (C) World map of scientific collaboration between countries, highlighting the global distribution of research activity. (D) Keyword co‐occurrence network, showing the main thematic clusters associated with green onion quality.

Figure 2 shows the results of the bibliometric analysis carried out on literature created on *A. fistulosum* , based on scientific data collected worldwide through data science analysis, highlighting the main terms, trends, and thematic networks related to the quality of this product. In the word cloud (Figure 2A), the most frequently used terms are “onion and quality”, followed by the word “soil”, one of the most studied topics in recent years given its relationship with pathogens that attack this crop. Words such as “phenolic” “chlorophyll” “volatile” and “bioactive” stand out, suggesting an interest in chemical compounds that modify the biochemical, nutritional, and sensory value of green onions. Likewise, it highlights the presence of words such as “storage”, “drying”, “moisture” and “treated” which are key terms in the post‐harvest stage of this product.

The results of the thematic trends studied since 2001 (Figure 2B) indicate that, starting in 2011, there was a sustained increase in the frequency of scientific publications related to quality parameters for this species, with a significant peak in 2012. While core terms such as “quality”, “vegetables” and “onion” are constantly present, there has been a notable gradual incorporation of complementary keywords that reveal an increasingly complex and multidimensional treatment of the concept of quality. Terms such as “growth”, “yield” “chlorophyll”, “storage”, “antioxidant” and “volatile” point to specific dimensions of quality such as nutritional, production, post‐harvest, and sensory attributes of the product, a pattern that indicates a shift in the scientific approach from a focus on agronomic performance to a comprehensive analysis that combines physical, chemical, bioactive, and perceptual attributes.

Since 2020 (Figure S1A), terms such as “antioxidants”, linked to the biological and functional activity of the product, have been used more frequently, along with new analytical methodologies such as “HPLC” “GC–MS” and “hyperspectral analysis” used to characterize bioactive compounds. Furthermore, in 2024, topics such as precision agriculture, management of physiological stress caused by high temperatures, and food safety have emerged, which not only broaden the technical spectrum of onion research but also reinforce the idea of quality as a concept that encompasses everything from production conditions to the nutritional and health impact of food.

Scientific collaboration between countries (Figure 2C) reveals a globally distributed research landscape on *A. fistulosum* quality. East Asia emerges as the main center of scientific production, with China leading in publication volume and number of prominent authors (Figure S1B,C), followed by Japan and South Korea, while significant contributions are also observed from North America, particularly the United States, and from North Africa, with Egypt playing a notable role. This distribution highlights the global relevance and international scope of the analyzed scientific corpus, reflecting a geographically diverse research landscape on *A. fistulosum* . Research activity is strongly concentrated in regions with well‐established horticultural science systems, where advances in post‐harvest management, bioactive compound characterization, and sensory quality assessment have driven sustained scientific output. These regions act as major hubs for methodological development and thematic innovation, contributing to globally shared knowledge on quality attributes. Within South America, Colombia stands out for its collaborative activity, contributing mainly from an applied perspective linked to production systems and market‐oriented quality assessment, and complementing the broader global research framework.

The co‐occurrence map was created from keywords in the documents consulted (Figure 2D), where each node represents a keyword and its size reflects the frequency with which it appears in the literature, grouping the terms into clusters differentiated by color. One of the main nodes is product quality, which is in turn related to terms such as “color” “texture” and “variety” demonstrating that quality has been approached from directly observed structural and sensory attributes. This node is also related to concepts such as “market” and “consumer” as well as “fresh produce” and “innocuity” suggesting that quality is considered not only in terms of the physical properties of the product, but also in terms of consumer perception and safety for consumption. Other terms related to quality dimensions include “bioactive compound”, “quercetin” and “antioxidant capacity” which are related to functional and nutritional quality, as well as “storage” and “yield” which are linked to post‐harvest stability and productive yield.

### 3.2 Extraction of Quality Parameters for Green Onion Using NLP

#### 3.2.1 Evaluation of Physical and Chemical Quality Parameters of Green Onions in Post‐Harvest

The meta‐analysis identified a set of physical, chemical, and functional parameters that structure the concept of quality in green onion from a multidimensional perspective. First, physical attributes such as pseudostem weight and diameter define the structural quality of the product. The Agrosavia Aquitania 1 and Agrosavia Tota 1 varieties have average weights of 114.9 and 106.5 g, respectively, and diameters ranging from 1.79 to 1.66 cm (AGROSAVIA 2025; Galindo Pacheco 2020).

From the chemical component, variables that contribute to nutritional quality and preservation were identified. The moisture content recorded at 12% for both varieties and dry matter with values between 7.43% and 7.92% have decisive impacts on the texture, resistance to damage, and shelf life of the product. The levels of pyruvic acid, at 30.4 and 31.5 μmol/g dry weight, indicate a high concentration of sulfur compounds responsible for the characteristic flavor, as well as bioactive properties associated with health.

In terms of functional compounds, sulfides, flavonoids, and phenols emerge as key indicators of the functional quality of green onion. The concentrations reported in the literature vary between 0.9 and 5 mg/100 g for sulfides, and up to 56 mg/100 g for flavonoids. These compounds enrich the nutritional profile of the product and reinforce its antioxidant, antimicrobial, and therapeutic potential, providing added value from the perspective of health‐conscious consumers.

Complementary studies address additional dimensions of the concept of quality. For example, Wang, Qiao, et al. (2023) identified aromatic compounds responsible for sensory attributes such as aroma and flavor, which are fundamental to product acceptance. Dong et al. (2013) and Murugesan et al. (2011) and demonstrated how agronomic management, temperature, and irradiation influence texture, color, safety, and security, which are key aspects of post‐harvest quality. In turn, Yaguchi et al. (2009) highlighted the presence of fructans and reducing sugars that improve the flavor and nutritional value of the product.

Finally, post‐harvest handling is also recognized as a determining factor in final quality. Harvesting, cleaning, storage, and handling practices affect product stability, presentation, and safety—aspects that are closely related to consumer perception of quality.

#### 3.2.2 Main Bioactive Compounds Reported in Green Onions as Multidimensional Quality Parameters

**FIGURE 3:**
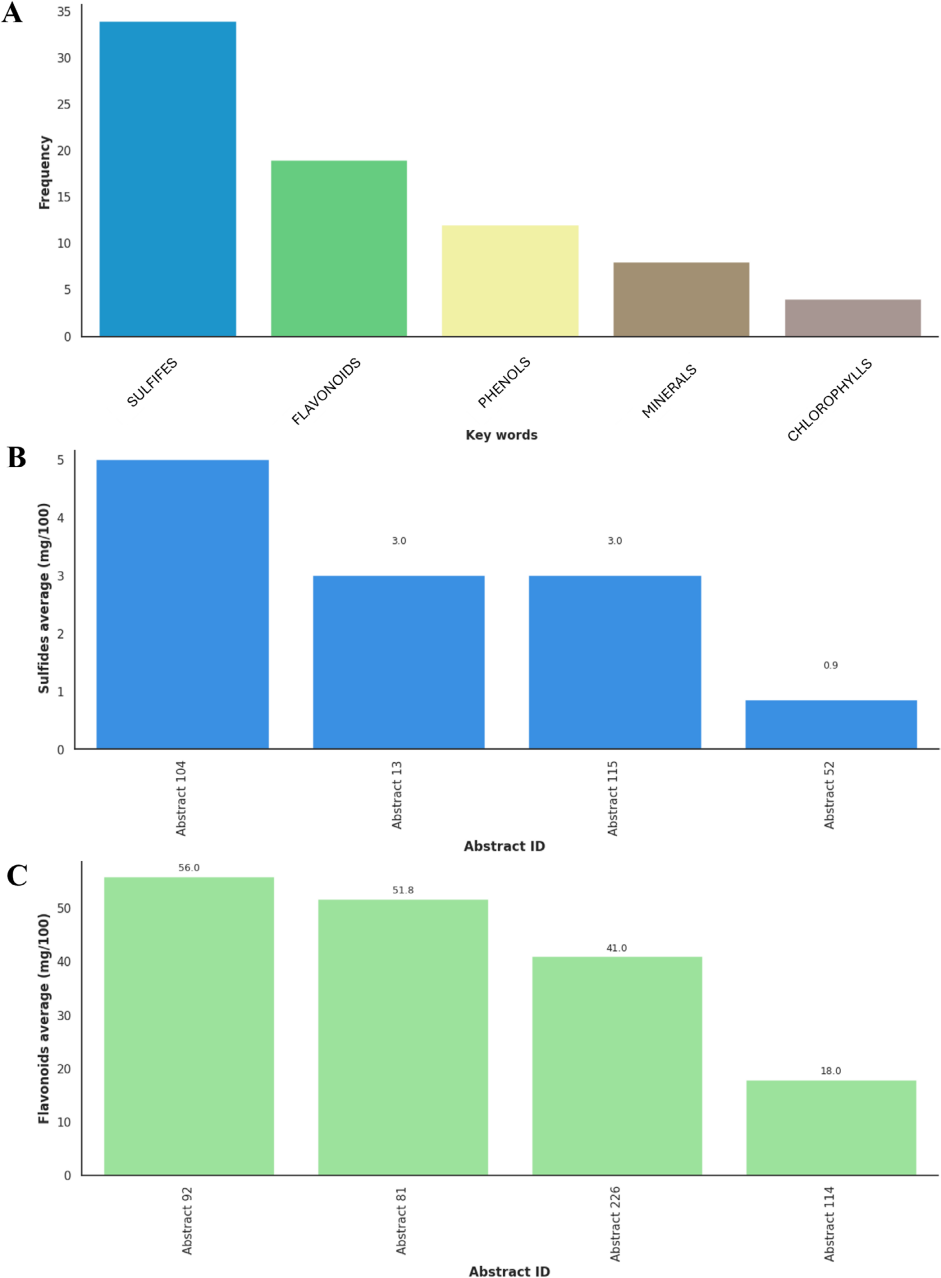
Extraction of keywords and concentrations or abundances of compounds with bioactive potential from green onion. (A) Frequency of occurrence of the main compounds groups in the analyzed abstracts. (B) Average concentration of sulfides reported in selected studies (mg/100 g), highlighting their relevance to functional and sensory quality. (C) Average concentration of flavonoids reported in bibliographic material (mg/100 g), reflecting their contribution to nutritional and biofunctional attributes.

**FIGURE 4:**
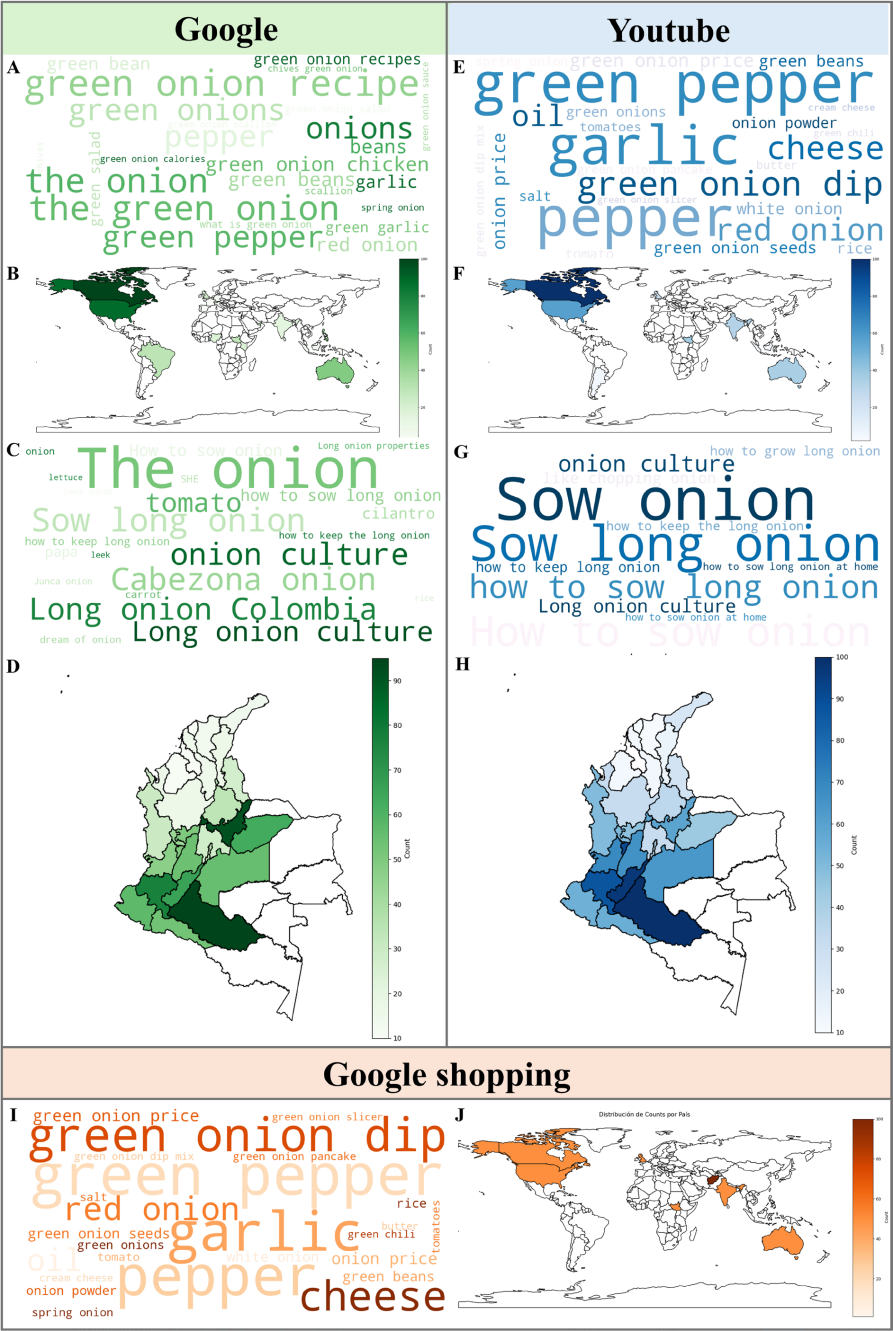
Analysis of search trends for the term “green onion” using Google Trends across three digital platforms at global and national (Colombia) scales. Google: (A) word cloud of the most frequent associated search terms worldwide and (B) global map showing the geographical distribution of search interest. (C) word cloud of associated terms at the national level and (D) national map of search distribution in Colombia. YouTube: (E) global word cloud of associated search terms and (F) global map of search distribution. (G) national word cloud and (H) national map for Colombia. Google Shopping: (I) global word cloud of associated search terms and (J) global map showing the geographical distribution of search interest.

Figure 4 presents the results obtained in the meta‐analysis on the bioactive compounds most frequently reported in the literature, currently considered as multidimensional quality parameters in green onion. Figure 3A represents the frequency of mentions of different bioactive compounds in the abstracts of the studies analyzed. Sulfides are the most frequently mentioned compounds, indicating their relevance in studies on green onion, possibly due to their impact on the aroma and sensory quality of the product. Secondly, flavonoids and phenols also appear frequently, being notable for their antioxidant properties and their influence on the nutritional quality of onions. To a lesser extent, minerals and chlorophylls are mentioned, suggesting that although they are important, they are not the main focus in the literature.

Regarding the specific values of the compounds, Figure 3B represents the average total sulfur compounds expressed in milligrams per 100 g of fresh sample (mg/100 g). The study identified as Abstract 104 reports the highest concentration with 5 mg/100 g of total sulfur compounds, mainly allicin, a metabolite responsible for the characteristic aroma of green onions and their antimicrobial and antioxidant activity. The studies identified as Abstract 13 (Abdelrahman et al. 2019) and Abstract 115 present intermediate concentrations of 3 mg/100 g of allicin and thiosulfinate derivatives, also linked to cardiovascular and functional benefits.

Abstract 52 (Yang et al. 2017) reports the lowest concentration with 0.9 mg/100 g of sulfur compounds, although in this case the study highlights a high presence of other bioactive metabolites such as total phenols, flavonoids, and phytosterols, which compensate for the low concentration of sulfides with a high total antioxidant capacity. On the other hand, Figure 3C shows the average flavonoid content in the studies, with values that vary considerably. The study identified as Abstract 92 reports the highest concentration of flavonoids found, representing 56 mg quercetin/100 g. The studies identified as Abstract 81 and Abstract 226 report total flavonoid concentrations of 51.8 and 41 mg/100 g, respectively; however, the study identified as Abstract 114 reports a value of 18 mg/100 g of total flavonoids. It is important to emphasize that the values obtained through NLP depend directly on the particularities of each study analyzed.

Beyond their reported concentrations, sulfides and flavonoids identified across the analyzed studies have been consistently associated with clinically relevant health effects (Xi et al. 2025). Recent clinical and intervention studies have linked dietary flavonoids, particularly quercetin, to improvements in oxidative stress markers, endothelial function, and inflammatory status in adults (Cassidy et al. 2011; Raman et al. 2019), while organosulfur compounds have been associated with cardiometabolic and immunomodulatory benefits (Putnik et al. 2019; Vazquez‐Prieto and Miatello 2010). From a quality perspective, these compounds are directly linked to attributes that define quality along the value chain, including oxidative stability, visual integrity, and shelf‐life performance. Consequently, the ranges identified provide a reference to interpret how agronomic conditions and postharvest handling may modulate biofunctional quality in Phase 4, and how these biochemical traits ultimately translate into consumer valued attributes addressed in Phase 5.

### 3.3 Web Trend Analysis on Green Onion

The analysis of digital trends shown in Figure 4 allows us to identify how the quality of green onion is perceived and valued by the general public through platforms such as Google, YouTube, and Google Shopping. Unlike technical and scientific approaches, these non‐specialized searches reveal practical, commercial, and mainly sensory dimensions that define the everyday experience with the product and complement its technical characterization.

In general terms, queries on these three platforms revolve around functional, sensory, and usage attributes, reflecting consumer interest in aspects such as taste, culinary versatility, presentation, preservation, and commercial value. On Google (Figure 4A–D), terms such as “green onion recipe”, “green onion chicken”, and “green onion sauce” position gastronomic use as one of the most important factors in building quality. This orientation highlights the sensory dimension, where flavor and aroma become key references. In addition, the presence of terms such as “green garlic”, “green pepper”, and “scallion” suggests a comparison and association with other ingredients, which strengthens the perception of the product as versatile and functional.

On YouTube (Figure 4E–H), searches such as green onion dip, green onion pancake, and green onion seeds continue to reinforce the importance of its use in specific preparations and in establishing cultivation. Quality here is expressed both in terms of sensory experience and ease of access to practical information, especially in domestic contexts. This platform also shows the value of the product from a structural and post‐harvest perspective, especially in producing countries such as Colombia, where searches are related to its preservation.

On Google Shopping (Figure 4I,J), users search for terms such as “green onion seeds”, “green onion price”, “onion powder”, and “green onion dip”, revealing a commercial and product presentation focus. Quality in this context is related to its availability in different formats and its perceived economic value, showing that the perception of quality is also built on access, presentation, and price.

For its part, the spatial distribution of these searches across different platforms confirms that quality is a variable concept that depends on geographical context (Ospina‐Sanchez et al. 2025). Globally, countries such as the United States and Canada show a pattern focused on the culinary and commercial use of the product, prioritizing functionality in the kitchen, availability in stores, and its integration with other ingredients. In contrast, in Colombia, especially in regions such as Cundinamarca, Boyacá, and Caquetá, the varietal identity of the material, its cultivation cycle, and its role in local agriculture are valued.

### 3.4 Quality Parameters of Green Onion Evaluated Under Real Production and Harvesting Conditions

**FIGURE 5:**
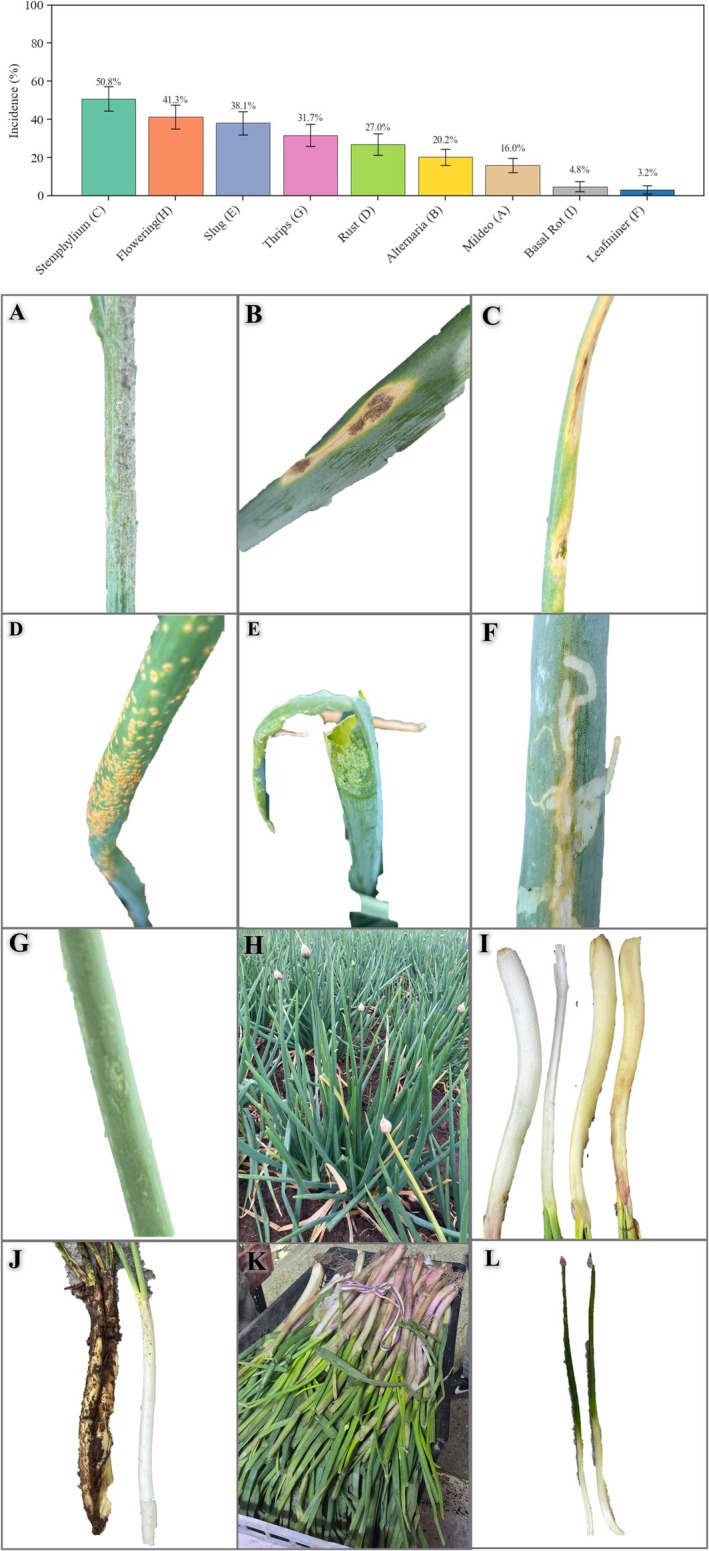
Incidence of phytosanitary problems observed in onion crops during the harvest period in the municipality of Aquitania, Boyacá, Colombia. (A) Downy mildew caused by *Peronospora destructor*. (B) Alternaria caused by *Alternaria porri*. (C) Leaf blight caused by *Stemphylium vesicarium*. (D) Onion rust causesture. (K) Pseudostems of green onion with purple‐reddish discoloration. (L) Rootless pseudostems of green onion. d by *Puccinia alli*. (E) Damage caused by slugs. (F) Galleries made by the leaf miner fly *Liriomyza huidobrensis* . (G) Damage caused by thrips (*Thrips tabaci, Frankliniella occidentalis*). (H) Plants in bloom (spiking). (I) Basal rot caused by *Fusarium* spp. (J) Pseudostem damaged by excess moisture. (K) Green onion pseudostems with purple‐reddish coloration. (L) Green onion pseudostems without root.

During the analysis, under real harvesting conditions, multiple biotic and physiological factors were identified that directly affect onion quality in its different dimensions (Figure 5). Among the main findings, the presence of “downy mildew” caused by the fungus *Peronospora destructor* stands out (Figure 5A), a disease that weakens the plant by reducing the functional photosynthetic area, which results in reduced pseudostem development, affecting its weight, caliber, and consistency. Leaf diseases such as “Alternaria” caused by the fungus *Alternaria porri* (Figure 5B) and “leaf blight” caused by *Stemphylium vesicarium* (Figure 5C) cause necrosis and desiccation of leaves, affecting visual quality, reducing shelf life, and decreasing acceptance in the fresh market. Similarly, “onion rust” caused by the fungus *Puccinia allii* (Figure 5D) causes orange spots and pustules, damaging the visual uniformity of the crop, with direct implications for its aesthetic appearance and commercial classification.

On the other hand, damage caused by slugs (Figure 5E) was observed, which causes irregular perforations in the leaves, compromising the visual appearance and safety of the product by facilitating the entry of secondary pathogens. The galleries of the leaf miner *Liriomyza huidobrensis* (Figure 5F) also affect the integrity of the foliage, weakening the plant and affecting the development of the pseudostem, which results in reduced yield and visible physical defects in the final product.

The dual presence as *Thrips tabaci* and *Frankliniella occidentalis* (Figure 5G) poses a double risk to quality because, on the one hand, they cause silver spots and deformities that alter the color and surface texture of the leaves, and on the other, they are vectors of viruses, compromising the health quality of the crop. Premature flowering or heading (Figure 5H) is another factor that severely affects sensory and structural quality, as it induces lignification of the pseudostem, making it more fibrous and less tender. Figure 5I illustrates the critical basal rot caused by *Fusarium* spp., which directly deteriorates the pseudostem, the organ consumed, generating post‐harvest losses, reducing shelf life, and seriously compromising product safety. These losses pose a direct threat to the sustainability of the production system and to the end consumer's perception of quality.

### 3.5 Variables That Affect the Quality of Green Onion for Distribution and Consumption

The survey on green onion consumption received a total of 115 responses as of July 31, 2025. In terms of geographical origin, participants were mainly distributed in Cundinamarca and Antioquia, Colombia. This regional representation allows us to understand how perceptions of quality vary according to the areas of most frequent consumption. Regarding the profile of the participants, 113 people identified themselves as consumers, while only 2 were producers, which places these results from the perspective of the end user. These results should be interpreted as exploratory, considering the non‐probabilistic nature and potential regional bias of the survey.

**FIGURE 6:**
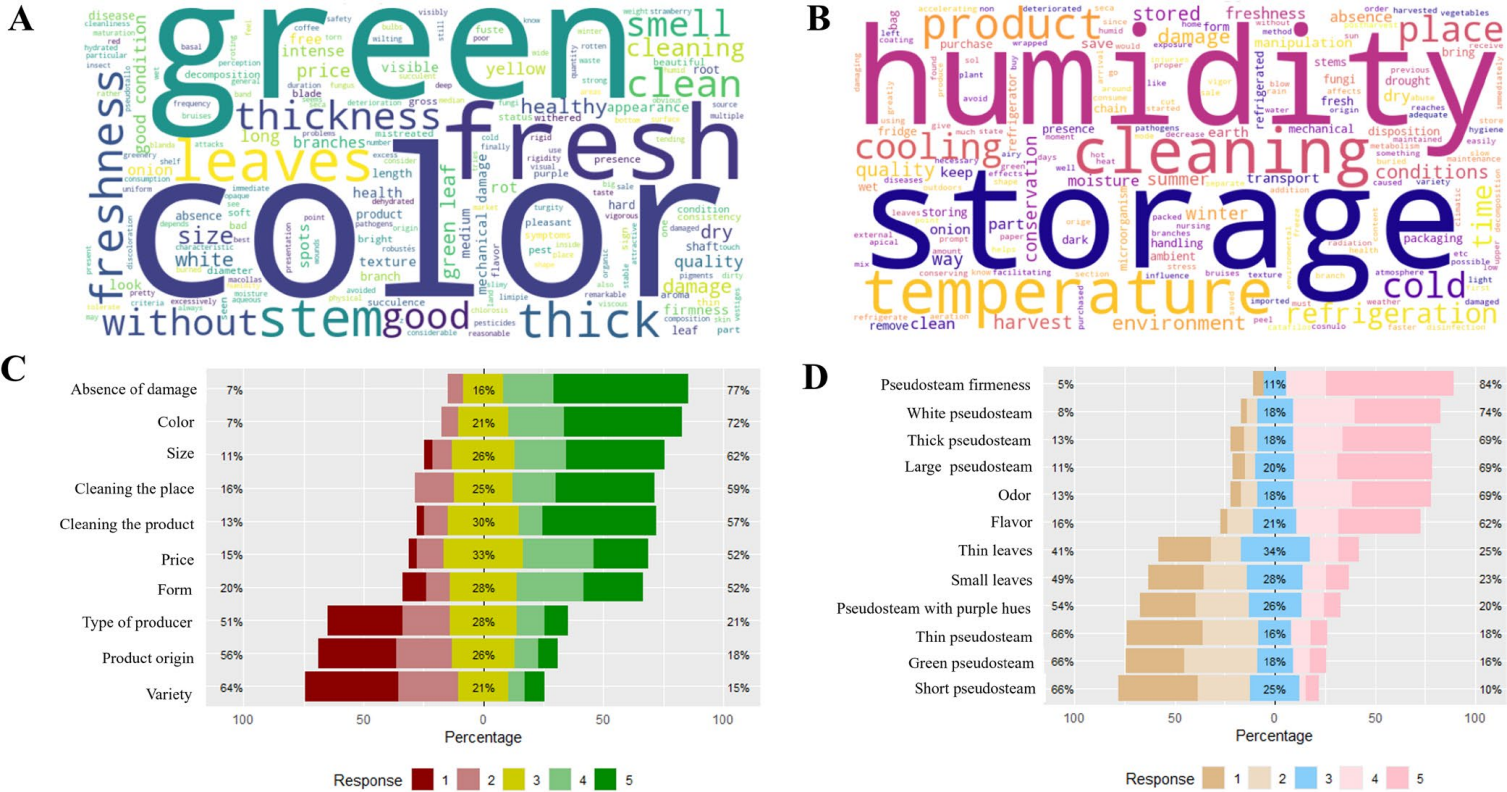
Consumer perception of green onion quality and post‐harvest attributes based on survey responses. (A) Word cloud representing the main product quality criteria mentioned by respondents. (B) Word cloud of factors perceived to influence product shelf life. (C) Consumer purchasing criteria expressed as the relative importance of different quality attributes. (D) Factors influencing post‐harvest quality of green onions as perceived by consumers.

The consumer perception analysis identified the most valued attributes in defining the quality of green onion, integrating sensory, structural, and health aspects. Through the word cloud generated from open‐ended responses (Figure 6A), it can be seen that the most frequent terms are “color”, “green”, “fresh”, “without damage”, “clean”, “stem” and “thickness”. Regarding the factors affecting post‐harvest conservation (Figure 6B), the most prominent keywords were “storage”, “temperature”, “humidity” and “cleaning”, reflects a clear consumer perception of handling practices and environmental conditions as key determinants of quality preservation. Internationally, green onion is typically marketed fresh and exhibits a short shelf life of approximately 7–10 days at 10°C, under standard postharvest conditions, with quality rapidly declining at higher temperatures (University of California 2025). This perception is consistent with post‐harvest literature, which identifies temperature control, moisture management, hygienic handling, and appropriate storage conditions as critical factors influencing deterioration rates, visual quality retention, and shelf‐life performance in leafy green onion (Aguilar Aguilar 2016; Sánchez et al. 2019).

The results obtained using the Likert scale (Figure 6C) indicate that the attributes with the highest level of perceived importance were absence of damage (77%), color (72%), size (69%), and cleanliness of the premises (59%). These attributes represent key components in the purchase decision, closely linked to the perception of safety, freshness, and presentation of the food. Notably, these consumer valued attributes directly mirror physicochemical and postharvest quality parameters reported in the literature and observed under field conditions, reinforcing the linkage between measurable quality traits and perceived product value.

Finally, the analysis of specific preferred attributes (Figure 6C) showed that the firmness of the pseudostem (84%) and its white color (74%) are the traits most valued by consumers. Attributes such as “thick” and “long pseudostems” as well as “large leaves” also obtained high levels of preference (> 60%), while characteristics such as “*short pseudostem”*, “green pseudostem”, “thin pseudostem” and “purple hues” were poorly accepted (< 30%) (Figure 6D). These results confirm that the perception of quality in green onion is strongly influenced by visual and structural criteria, which is consistent with their nature as a fresh product for immediate consumption. Furthermore, their acceptance in the market depends both on intrinsic factors, such as firmness and pseudostem color, and extrinsic factors, including commercial presentation, cleanliness, and observable sanitary condition at the time of purchase.

## 4 Discussion

Through an interdisciplinary approach, this study addresses a critical knowledge gap arising from the absence of a unified baseline to define green onion quality from a multidimensional perspective. While previous research has largely focused on isolated physicochemical and biofunctional attributes (Demartelaere et al. 2020; Kurnia et al. 2021), these efforts have typically relied on reductionist frameworks that fail to capture the complex interactions among biological, technical, environmental, and particularly social dimensions of quality (Cha et al. 2008; Wang, Qiao, et al. 2023). Although defining multidimensional quality in agricultural products is inherently complex and subjective due to the interplay of biological, technical, cultural, and perceptual factors (Ospina‐Sanchez et al. 2025; Ramírez‐Gil et al. 2019), our results demonstrate that this complexity can be effectively addressed through an integrated, multi‐phase methodological framework. Through the integration of evidence from scientific literature, meta‐analysis of physicochemical and biofunctional parameters, digital trend analysis, field observations, and consumer perception, we identified a coherent and reproducible set of quality determinants that provides a systemic, data‐driven foundation for understanding green onion quality.

Our results indicate that the multidimensional quality of green onion converges around a limited and consistent set of core attributes across the value chain. These include visual and structural traits such as freshness, pseudostem thickness, firmness, uniformity, and absence of visible damage; biofunctional components, particularly sulfur compounds and flavonoids that underpin sensory and nutritional value; and sanitary and postharvest conditions that directly determine shelf life and market suitability. Consumer perception plays an integrative role by validating these attributes and translating measurable and observable traits into acceptance criteria at the point of purchase. The observed convergence between objective quality parameters and consumer perception underscores how physicochemical and postharvest traits are effectively transformed into consumer‐valued attributes. Under this integrative perspective, green onion quality can be defined as a multidimensional construct emerging from the alignment of physicochemical, sensory, and biofunctional attributes, sanitary and postharvest conditions, visual and structural characteristics, and consumer‐valued criteria, all shaped by territorial, productive, and cultural contexts.

Our findings reinforce that the identification and quantification of bioactive compounds represent a central component of contemporary quality frameworks, as they underpin robust phytochemical characterization with direct nutritional, functional, and commercial relevance (Ajayi et al. 2019; Yan et al. 2023). In green onion, compounds such as flavonoids and sulfides play a decisive role in shaping nutritional value, sensory attributes, and postharvest shelf life (Altemimi et al. 2017; Ijeomah et al. 2020). Beyond their physiological effects, these metabolites support emerging value propositions aimed at product differentiation and the development of biofunctionally targeted foods, particularly those aligned with healthy aging and longevity trends (Hossain et al. 2025; Hu and Yan 2025). The compound concentrations observed in this study fall within internationally reported ranges, although methodological variability among studies partly explains observed differences (Anand David et al. 2016). Collectively, these results point to a distinctive phytochemical profile with strong potential for differentiation in specialized markets and for the development of origin‐based quality schemes, as demonstrated in other agri‐food products (Kim et al. 2015; Venu et al. 2019).

One key finding of our study is that quality significantly varies by region, influenced by culinary use, visual presentation, and the product's dietary role. Although this study centers on the Andean context, similar quality perceptions have been observed in other major green onion producing regions, indicating context‐dependent patterns. In Colombia, emphasis is placed on sensory characteristics such as the pseudostem's whiteness, thickness, and freshness, while other countries prioritize functional components or nutritional value. This underscores the necessity of integrating geographical and cultural factors into the quality concept, particularly for crops with local significance, alongside methods that facilitate quality interpretation across diverse cultural and production contexts (Datta et al. 2020; Kyriacou and Rouphael 2018). From a methodological perspective, our multi‐source and multi‐tool approach demonstrates that the integration of heterogeneous data supported by robust analytical methods such as bibliometrics, NLP, and data‐driven synthesis enables a meaningful approximation toward the unification of multidimensional quality for green onion, while simultaneously revealing structural weaknesses across the value chain. Despite growing recognition of the importance of quality integration, effective information flow among value chain actors remains one of the most critical challenges, largely due to limited coordination, poor data sharing, and fragmented decision‐making processes (Ahoa et al. 2025; Vahdanjoo et al. 2025). Evidence from collaborative initiatives documented by the USDA and the World Bank indicates that shared digital platforms can strengthen quality practices, improve operational efficiency, and reduce uncertainty by enabling feedback loops in which consumer data inform production and logistical decisions (Chai et al. 2021; Reynolds 2023).

In this context, our bibliometric, data trends, NLP‐based, productive data, and consumer perceptions analyses reveal substantial conceptual and methodological fragmentation in the literature on green onion quality, driven by the absence of standardized assessment protocols, the coexistence of diverse disciplinary perspectives, and strong territorial specificity in production and consumption systems (Čepulienė et al. 2024; Hwang et al. 2020). This dispersion is due to factors such as (i) the absence of standardized technical protocols for assessing vegetable quality, (ii) the diversity of disciplinary approaches to determining quality, ranging from agronomy and food chemistry to nutrition and social sciences, and (iii) differences in production and commercial contexts, given that green onion has production and consumption patterns closely linked to specific territories. Similar challenges have been reported for other territorially rooted crops, such as tomato and potato, where heterogeneous methodologies hinder comparability and consensus on quality standards (Anisimov 2021; Panthee et al. 2013). Although global research on physicochemical and biofunctional attributes shows consistent patterns, regional analyses highlight that these parameters are interpreted and prioritized differently according to cultural, agronomic, and market contexts a distinction that is particularly relevant for *A. fistulosum* , whose value is deeply embedded in Andean food systems, local culinary traditions, and rural economies (Arzeno and Troncoso 2012; Ospina‐Sanchez et al. 2025).

Our study opens new research avenues aimed at standardizing quality parameters for green onion and other vegetables through the application of modern analytical tools and data‐integration approaches (Čepulienė et al. 2024; Martínez‐Saldarriaga et al. 2025). Strengthening field‐level data collection under unified criteria and complementing existing open‐access platforms is essential to integrate scientific, technical, and commercial information in an accessible and continuously updated manner. Moreover, bridging academic knowledge with the realities of the productive sector remains a priority. Actively involving farmers, technicians, and market actors in quality assessment processes can enhance the relevance, applicability, and sustainability of the proposed criteria, support the validation of interdisciplinary approaches under real production conditions, and facilitate the development of differentiated markets based on value‐added quality attributes (Čepulienė et al. 2024; Ciaccia et al. 2019).

The proposed framework translates the information generated in this study into operational criteria that can serve as concrete references for quality standardization schemes, certification processes focused on differentiated plant materials, and technical extension strategies, thereby strengthening decision‐making across the green onion value chain. Beyond its immediate applicability, this work highlights the need to consolidate and refine the concept of green onion quality by addressing several key challenges. These include improving the precision of multidimensional quality definitions, developing indirect and rapid measurement alternatives capable of capturing complex quality attributes, and identifying more effective tools for preserving quality factors that are highly susceptible to rapid deterioration during postharvest handling. Addressing these limitations is particularly critical given that many of the attributes defining green onion quality are dynamic and short‐lived, directly influencing shelf life, marketability, and consumer acceptance. In addition, future research should also explore how climate change‐related scenarios influence the physicochemical, biofunctional, and perceptual expression of green onion quality along the value chain, further reinforcing the strategic value of this crop (Moretti et al. 2010).

## 5 Conclusion

A baseline was established for evaluating the quality of green onion, addressing a critical knowledge gap related to the fragmentation of quality criteria and their interpretation across the value chain. Green onion quality is influenced by multiple parameters such as the firmness and color of the pseudostem, functional attributes, including flavonoid content (38.4 and 73.3 mg of quercetin/100 g of fresh weight), sulfur compounds (71.6 and 353.6 μmol/g of fresh weight), and the absence of visible damage. From a perceptual standpoint, consumers prioritized attributes such as thickness (89.3%), product freshness (93.6%), and absence of damage (87.1%), demonstrating that the perception of quality is strongly influenced by visual and sensory factors, as well as cultural roots and context of use. The concept of quality for green onion is defined as a multidimensional construct emerging from the alignment of physicochemical sensory and biofunctional attributes, sanitary and postharvest conditions, visual and structural characteristics, and consumer‐valued criteria, all shaped by territorial, productive, and cultural contexts. This study reinforces the need to incorporate the geospatial and cultural dimensions as structural components in the definition of quality for fresh‐consumption crops with a strong regional identity, such as green onion. Involving indirect sources such as academic literature and digital platforms, and direct sources such as agronomic observation and consumer surveys, provided a holistic understanding of the product, allowing us to map the current state of knowledge, highlighting gaps in knowledge, regional contrasts, and opportunities for standardizing quality criteria.

## Author Contributions

**Leslie Walessa Castaño‐Tarazona:** conceptualization (equal), data curation (equal), formal analysis (equal), investigation (equal), methodology (equal), project administration (equal), resources (equal), software (equal), supervision (equal), validation (equal), visualization (equal), writing review and editing (equal). **Juan Camilo Henao‐Rojas:** conceptualization (equal), funding acquisition (equal), investigation (equal), methodology (equal), project administration (equal), resources (equal), supervision (equal), validation (equal), visualization (equal), writing review and editing (equal). **Joaquín Guillermo Ramírez‐Gil:** conceptualization (equal), formal analysis (equal), investigation (equal), methodology (equal), resources (equal), software (equal), supervision (equal), validation (equal), visualization (equal), writing review and editing (equal).

## Funding

Sistema General de Regalias (SGR) de Minciencias Colombia, through the project “Mejoramiento del sistema productivo de cebolla de rama enfocado a las demandas del mercado en fresco y/o agroindustria en el departamento de Antioquia” (BPIN code: 2020000100413).

## Ethics Statement

This study was conducted in strict adherence to ethical standards and in full compliance with Habeas Data regulations. Informed consent was obtained for each survey, and participants were clearly informed that their data would be treated with the utmost confidentiality. All procedures were carried out following ethical guidelines to ensure privacy, transparency, and responsible data management.

## Conflicts of Interest

The authors declare no conflicts of interest.

## Supporting information


**Figure S1:** Thematic and geographic analysis of scientific production on *Allium fistulosum* L. (2001–2025). (A) Evolution of research topics related to green onion quality. (B) Distribution of most productive authors by country. (C) Timeline of scientific productivity per author.

## Data Availability

In order to guarantee the transparency, reproducibility, and replicability of our work, we have shared the data, codes and other information to support our work. Users may consult and make use of the information and analysis scripts in the following repository, which we suggest be cited (the paper) if the information, part of it, or the codes are used. The repository is free and accessible in the following direction: https://github.com/agrocompuepidemlab/Multidimensional‐perspective‐on‐the‐quality‐of‐Green‐Onion.git.

## References

[fsn371565-bib-0002] Abdelrahman, M. , S. Hirata , Y. Sawada , et al. 2019. “Widely Targeted Metabolome and Transcriptome Landscapes of *Allium fistulosum*–*A. Cepa* Chromosome Addition Lines Revealed a Flavonoid Hot Spot on Chromosome 5A.” Scientific Reports 9, no. 1: 3541. 10.1038/s41598-019-39856-1. 30837538 PMC6400954

[fsn371565-bib-0003] AGROSAVIA . 2025. “Variedades de cebolla de rama.” https://www.agrosavia.co/productos‐y‐servicios/modelos‐productivos/modelo‐productivo‐de‐cebolla/manejo‐agronómico/variedades‐de‐cebolla‐de‐rama/.

[fsn371565-bib-0004] Aguilar Aguilar, P. A. 2016. “Modelo tecnológico para el cultivo de cebolla de rama *Allium fistulosum*, en el departamento de Antioquia.” Corporación colombiana de investigación agropecuaria ‐ AGROSAVIA.

[fsn371565-bib-0005] Ahoa, E. , A. Kassahun , C. Verdouw , and B. Tekinerdogan . 2025. “Challenges and Solution Directions for the Integration of Smart Information Systems in the Agri‐Food Sector.” Sensors 25, no. 8: 2362. 10.3390/s25082362. 40285052 PMC12031551

[fsn371565-bib-0006] Ajayi, G. , M. Akinsanya , A. Agbabiaka , K. Oyebanjo , T. Hungbo , and J. Olagunju . 2019. “D‐Limonene: A Major Bioactive Constituent in *Allium fistulosum* Identified by GC‐MS Analysis.” Journal of Phytopharmacology 8: 257–259. 10.31254/phyto.2019.8509.

[fsn371565-bib-0007] Altemimi, A. , N. Lakhssassi , A. Baharlouei , D. G. Watson , and D. A. Lightfoot . 2017. “Phytochemicals: Extraction, Isolation, and Identification of Bioactive Compounds From Plant Extracts.” Plants 6, no. 4: 4. 10.3390/plants6040042. 28937585 PMC5750618

[fsn371565-bib-0008] Anand David, A. V. , R. Arulmoli , and S. Parasuraman . 2016. “Overviews of Biological Importance of Quercetin: A Bioactive Flavonoid.” Pharmacognosy Reviews 10, no. 20: 84–89. 10.4103/0973-7847.194044. 28082789 PMC5214562

[fsn371565-bib-0009] Anisimov, B. V. 2021. “Quality Standards for Various Categories of Potato Seed: Crop Surveys and Appraisement of Plantings and Tuber Analysis.” In Potato Seed Production, edited by S. V. Zhevora and B. V. Anisimov , 111–117. Springer International Publishing. 10.1007/978-3-030-60762-3_11.

[fsn371565-bib-0010] Arzeno, M. , and C. A. Troncoso . 2012. “Alimentos tradicionales andinos, turismo y lugar: Definiendo la nueva geografía de la Quebrada de Humahuaca (Argentina).” Revista de Geografía Norte Grande 52: 71–90. 10.4067/S0718-34022012000200005.

[fsn371565-bib-0011] Balkrishna, A. , M. Chaudhary , H. Sharma , et al. 2023. “Phytochemistry, Pharmacology, and Medicinal Aspects of *Allium fistulosum* L.: A Narrative Review.” Journal of Applied Pharmaceutical Science 13, no. 10: 107–118. 10.7324/JAPS.2023.142822.

[fsn371565-bib-0012] Batterton, K. A. , and K. N. Hale . 2017. “The Likert Scale What It Is and How to Use It.” Phalanx 50, no. 2: 32–39.

[fsn371565-bib-0013] Bautista Peña, J. R. 2020. “La cebolla de rama (*Allium fistulosum* L.) como alternativa de diversificación de cultivos en el corregimiento de La Granja, municipio de Sucre Santander.” https://hdl.handle.net/20.500.14625/18610.

[fsn371565-bib-0014] Bautista‐Romero, L. V. , J. D. Sánchez‐Murcia , and J. G. Ramírez‐Gil . 2025. “Data Science for Pattern Recognition in Agricultural Large Time Series Data: A Case Study on Sugarcane Sucrose Yield.” Heliyon 11, no. 4: e42632. 10.1016/j.heliyon.2025.e42632. 40034300 PMC11874567

[fsn371565-bib-0095] Beretta, H. V. , and C. R. Galmarini . 2016. “Análisis bioquímicos, genéticos y metodológicos de compuestos bioactivos responsables del sabor y las propiedades funcionales en cebollas y otras especies consumibles de Aliáceas.” https://ri.conicet.gov.ar/handle/11336/93599.

[fsn371565-bib-0016] Carmona Bayonas, J. 2022. “Mejora en la producción y calidad de lechuga mediante la optimización de las condiciones de cultivo en sistemas hidropónicos bajo invernadero. Proyecto de investigación: Nuevos sistemas de cultivo para la producción de hortalizas de hoja.” https://digitum.um.es/digitum/handle/10201/122274.

[fsn371565-bib-0017] Cassidy, A. , É. J. O'Reilly , C. Kay , et al. 2011. “Habitual Intake of Flavonoid Subclasses and Incident Hypertension in Adults.” American Journal of Clinical Nutrition 93, no. 2: 338–347. 10.3945/ajcn.110.006783. 21106916 PMC3021426

[fsn371565-bib-0018] Castaño‐Tarazona, L. W. , L. S. Hernández‐Sánchez , L. A. Valbuena‐Gaona , H. E. Balaguera‐López , L. M. Melgarejo , and J. G. Ramirez‐Gil . 2025. “Approximation to the Concept of Multidimensional Quality of Blueberry Fruits From the High Colombian Tropics.” Journal of Agriculture and Food Research 24: 102504. 10.1016/j.jafr.2025.102504.

[fsn371565-bib-0019] Castaño‐Tarazona, L. W. , L. A. Valbuena‐Gaona , and J. G. Ramírez‐Gil . 2025. “Assessing the Impact of Epidermal Rotting on Postharvest Mango Quality: Utilizing Multidimensional Parameters and AI‐Based Methods for Damage Classification.” Measurement: Food 20: 100259. 10.1016/j.meafoo.2025.100259.

[fsn371565-bib-0020] Ceballos‐Sierra, F. , and S. Dall'Erba . 2021. “The Effect of Climate Variability on Colombian Coffee Productivity: A Dynamic Panel Model Approach.” Agricultural Systems 190: 103126. 10.1016/j.agsy.2021.103126.

[fsn371565-bib-0021] Čepulienė, V. , D. Juškevičienė , J. Viškelis , and R. Karklelienė . 2024. “Productivity and Quality Parameters of *Allium fistulosum* L.” Acta Agriculturae Scandinavica Section B Soil and Plant Science 74, no. 1: 2392506. 10.1080/09064710.2024.2392506.

[fsn371565-bib-0022] Cha, H.‐S. , A.‐R. Youn , S.‐H. Kim , J.‐W. Jeong , and B.‐S. Kim . 2008. “Quality Analysis of Welsh Onion (*Allium fistulosum* L.) as Influenced by Storage Temperature and Harvesting Period.” Korean Journal of Food Science and Technology 40, no. 1: 1–7.

[fsn371565-bib-0023] Chai, Q. , T. Nemecek , C. Liang , et al. 2021. “Integrated Farming With Intercropping Increases Food Production While Reducing Environmental Footprint.” Proceedings of the National Academy of Sciences of the United States of America 118, no. 38: e2106382118. 10.1073/pnas.2106382118. 34518225 PMC8463858

[fsn371565-bib-0024] Chergui, N. , and M. T. Kechadi . 2022. “Data Analytics for Crop Management: A Big Data View.” Journal of Big Data 9, no. 1: 123. 10.1186/s40537-022-00668-2.

[fsn371565-bib-0025] Choi, J. W. , M. Cho , K.‐S. Jung , J. H. Cho , J. H. Lee , and S. Lim . 2024. “Effects of Packaging Method and Root Trimming on Quality of Green Onion (*Allium fistulosum* L.) During Storage.” Food Science and Preservation 31, no. 3: 433–443. 10.11002/fsp.2024.31.3.433.

[fsn371565-bib-0026] Ciaccia, C. , M. Di Pierro , E. Testani , G. Roccuzzo , M. Cutuli , and D. Ceccarelli . 2019. “Participatory Research Towards Food System Redesign: Italian Case Study and Perspectives.” Sustainability 11, no. 24: 7138. 10.3390/su11247138.

[fsn371565-bib-0090] Civille, G. V. 1991. “Food Quality: Consumer Acceptance and Sensory Attributes.” Journal of Food Quality 14, no. 1: 1–8. 10.1111/j.1745-4557.1991.tb00044.x.

[fsn371565-bib-0027] Cruz, Y. S. B. , and J. C. A. Espinel . 2017. “Caracterización de la Cadena Productiva de la Cebolla de Rama y la Articulación Entre Productores en el Municipio de Aquitania Boyacá (2006–2014).”

[fsn371565-bib-0028] Datta, H. S. , G. Sharma , and S. S. Bora . 2020. “Geographical Indications in Horticulture: North East India Perspective.” International Journal of Current Microbiology and Applied Sciences 9, no. 1: 1207–1221. 10.20546/ijcmas.2020.901.134.

[fsn371565-bib-0029] Demartelaere, A. C. F. , W. Preston , H. A. F. Preston , et al. 2020. “Uso do hidrogel na família das aliaceas: *Allium fistulosum* e *Allium cepa*/Use hydrogel in Aliaceas family: Allium fistulosum and *Allium cepa* .” Brazilian Journal of Development 6, no. 11: 90411–90420. 10.34117/bjdv6n11-450.

[fsn371565-bib-0030] Dhal, S. B. , and D. Kar . 2025. “Leveraging Artificial Intelligence and Advanced Food Processing Techniques for Enhanced Food Safety, Quality, and Security: A Comprehensive Review.” Discover Applied Sciences 7, no. 1: 75. 10.1007/s42452-025-06472-w.

[fsn371565-bib-0031] Dong, Y. , Z. Cheng , H. Meng , H. Liu , C. Wu , and A. R. Khan . 2013. “The Effect of Cultivar, Sowing Date and Transplant Location in Field on Bolting of Welsh Onion (*Allium fistulosum*L.).” BMC Plant Biology 13, no. 1: 154. 10.1186/1471-2229-13-154. 24199907 PMC4226261

[fsn371565-bib-0032] Emam, M. S. 2009. “Impact of Packaging and Heat Treatment on Maintaining Green Onion Post Harvest Quality.” Egyptian Journal of Agricultural Research 87, no. 1: 245–257. 10.21608/ejar.2009.193151.

[fsn371565-bib-0033] Falcão, R. N. R. , M. Vrana , C. Hudek , et al. 2024. “Farmers' Perception of Soil Health: The Use of Quality Data and Its Implication for Farm Management.” Soil Use and Management 40, no. 1: e13023. 10.1111/sum.13023.

[fsn371565-bib-0034] Fallik, E. , and Z. Ilic . 2018. “Pre‐ and Postharvest Treatments Affecting Flavor Quality of Fruits and Vegetables.” In Preharvest Modulation of Postharvest Fruit and Vegetable Quality, edited by M. W. Siddiqui , 139–168. Academic Press. 10.1016/B978-0-12-809807-3.00006-8.

[fsn371565-bib-0035] Galeano Mendoza, C. H. , E. F. Baquero Cubillos , J. A. Molina Varón , and M. d. S. Cerón Lasso . 2018. “Agronomic Evaluation of Bunching Onion in the Colombian Cundiboyacense High Plateau.” International Journal of Agronomy 2018, no. 1: 4940589. 10.1155/2018/4940589.

[fsn371565-bib-0036] Galindo Pacheco, J. R. 2020. “Cebolla de rama (*Allium fistulosum* L.): Manual de recomendaciones técnicas para su cultivo en el departamento de Cundinamarca.” https://repository.agrosavia.co/bitstream/handle/20.500.12324/36816/Ver_documento_36816.pdf?sequence=1.

[fsn371565-bib-0037] Gisbert Mullor, R. 2023. “Análisis de la variabilidad genética del género capsicum frente a estreses abióticos para su uso como portainjertos.” Estudio de los mecanismos fisiológicos de tolerancia, del comportamiento agronómico y de la calidad del fruto, 1. Universitat Politècnica de València. http://purl.org/dc/dcmitype/text. https://dialnet.unirioja.es/servlet/tesis?codigo=326203.

[fsn371565-bib-0038] Hossain, M. S. , M. A. Wazed , S. D. Shuvo , et al. 2025. “Fortified and Functional Foods: Trends, Innovations, and Their Public Health Impact for Future Nutrient Enrichment.” Journal of Agriculture and Food Research 23: 102275. 10.1016/j.jafr.2025.102275.

[fsn371565-bib-0039] Hu, Y. , and Z. Yan . 2025. “Comprehensive Advances in Phytochemical Components, Bioactive Functionality, and Processing Applications of Mustard (*Brassica juncea* (L.) Czern.): A Review.” Frontiers in Nutrition 12: 1626333. 10.3389/fnut.2025.1626333. 40823021 PMC12350256

[fsn371565-bib-0040] Hwang, J. T. , J. A. Ryuk , H. J. Kim , D. H. Jung , and B. S. Ko . 2020. “Validation Study on the Geometric Isomers From Bulbs of Allium Fistulosum and Their Conversion.” Applied Biological Chemistry 63, no. 1: 37. 10.1186/s13765-020-00520-2.

[fsn371565-bib-0094] Ibaraki, T. , H. Ikeda , and H. Ohta . 1997. “Effects of Several Atmosphere Compositions on Keeping Quality of Welsh Onion (*Allium fistulosum* L.).” Food Preservation Science 23, no. 1: 3–7. 10.5891/jafps.23.3.

[fsn371565-bib-0041] Ijeomah, C. , O. Amuda , B. Babatunde , and P. Abutu . 2020. “Evaluation of Genetic Diversity of Spring Onions (*Allium fistulosum*) Based on DNA Markers.” Journal of Experimental Agriculture International 42: 23–33. 10.9734/jeai/2020/v42i330481.

[fsn371565-bib-0042] Jun, S.‐P. , H. S. Yoo , and S. Choi . 2018. “Ten Years of Research Change Using Google Trends: From the Perspective of Big Data Utilizations and Applications.” Technological Forecasting and Social Change 130: 69–87. 10.1016/j.techfore.2017.11.009.

[fsn371565-bib-0043] Kaur, G. 2024. “The Impact of Big Data Analysis on Trends.” Journal of Research in Science and Engineering 6, no. 7: 54–58. 10.53469/jrse.2024.06(07).09.

[fsn371565-bib-0044] Kayat, F. , A. Mohammed , and A. M. Ibrahim . 2021. “Spring Onion (*Allium fistulosum* L.) Breeding Strategies.” In Advances in Plant Breeding Strategies: Vegetable Crops: Volume 10: Leaves, Flowerheads, Green Pods, Mushrooms and Truffles, edited by J. M. Al‐Khayri , S. M. Jain , and D. V. Johnson , 135–182. Springer International Publishing. 10.1007/978-3-030-66969-0_4.

[fsn371565-bib-0045] Kim, D.‐O. , O. K. Chun , Y. J. Kim , H.‐Y. Moon , and C. Y. Lee . 2003. “Quantification of Polyphenolics and Their Antioxidant Capacity in Fresh Plums.” Journal of Agricultural and Food Chemistry 51, no. 22: 6509–6515. 10.1021/jf0343074. 14558771

[fsn371565-bib-0046] Kim, T. J. , K. B. Lee , S.‐A. Baek , et al. 2015. “Determination of Lipophilic Metabolites for Species Discrimination and Quality Assessment of Nine Leafy Vegetables.” Journal of Korean Society for Applied Biological Chemistry 58, no. 6: 909–918. 10.1007/s13765-015-0119-6.

[fsn371565-bib-0047] Kothari, D. , W.‐D. Lee , and S.‐K. Kim . 2020. “Allium Flavonols: Health Benefits, Molecular Targets, and Bioavailability.” Antioxidants 9, no. 9: 9. 10.3390/antiox9090888. PMC7555649 32961762

[fsn371565-bib-0048] Kumar, R. , S. Saxena , V. Kumar , V. Prabha , R. Kumar , and A. Kukreti . 2023. “Service Innovation Research: A Bibliometric Analysis Using VOSviewer Service Innovation.” Competitiveness Review 34, no. 4: 736–760. 10.1108/CR-01-2023-0010.

[fsn371565-bib-0049] Kurnia, D. , D. Ajiati , L. Heliawati , and D. Sumiarsa . 2021. “Antioxidant Properties and Structure‐Antioxidant Activity Relationship of Allium Species Leaves.” Molecules 26, no. 23: 23. 10.3390/molecules26237175. PMC8659087 34885755

[fsn371565-bib-0050] Kyriacou, M. C. , and Y. Rouphael . 2018. “Towards a New Definition of Quality for Fresh Fruits and Vegetables.” Scientia Horticulturae 234: 463–469. 10.1016/j.scienta.2017.09.046.

[fsn371565-bib-0092] Liu, L. , T. Jin , X. Li , Y. Zhu , and S. Song . 1999. “Non‐Destructive Quality Evaluation of Vegetables in China.” Acta Horticulturae 483: 245–250. 10.17660/ActaHortic.1999.483.26.

[fsn371565-bib-0051] Liverani, A. , G. Caligiana , L. Frizziero , D. Francia , G. Donnici , and K. Dhaimini . 2019. “Design for Six Sigma (DFSS) for Additive Manufacturing Applied to an Innovative Multifunctional Fan.” International Journal on Interactive Design and Manufacturing (IJIDeM) 13, no. 1: 309–330. 10.1007/s12008-019-00548-9.

[fsn371565-bib-0052] Maldonado Luna, S. M. 2012. “Manual Práctico Para El Diseño De La Escala Likert.” Xihmai 2, no. 4: 1–3. 10.37646/xihmai.v2i4.101.

[fsn371565-bib-0053] Martínez‐Saldarriaga, J. , J. C. Henao‐Rojas , D. H. Flórez‐Martínez , E. M. Cadena‐Chamorro , and D. P. Yepes‐Betancur . 2025. “Methodological Framework for Supporting Phytochemical Bioprospecting Re‐Search: A Case Study on Carrot ( *Daucus carota* L.) Crop By‐Products.” Heliyon 11, no. 3: e41822. 10.1016/j.heliyon.2025.e41822. 39916821 PMC11799957

[fsn371565-bib-0054] Mavragani, A. , G. Ochoa , and K. P. Tsagarakis . 2018. “Assessing the Methods, Tools, and Statistical Approaches in Google Trends Research: Systematic Review.” Journal of Medical Internet Research 20, no. 11: e9366. 10.2196/jmir.9366. PMC6246971 30401664

[fsn371565-bib-0055] Medina‐Jaramillo, C. , E. Gomez‐Delgado , and A. López‐Córdoba . 2022. “Improvement of the Ultrasound‐Assisted Extraction of Polyphenols From Welsh Onion (*Allium fistulosum*) Leaves Using Response Surface Methodology.” Food 11, no. 16: 2425. 10.3390/foods11162425. PMC9407235 36010426

[fsn371565-bib-0091] Mihafu, F. D. , J. Y. Issa , and M. W. Kamiyango . 2020. “Implication of Sensory Evaluation and Quality Assessment in Food Product Development: A Review.” Current Research in Nutrition and Food Science Journal 8, no. 3: 690–702. 10.12944/CRNFSJ.8.3.03.

[fsn371565-bib-0056] Moretti, C. L. , L. M. Mattos , A. G. Calbo , and S. A. Sargent . 2010. “Climate Changes and Potential Impacts on Postharvest Quality of Fruit and Vegetable Crops: A Review.” Food Research International 43, no. 7: 1824–1832. 10.1016/j.foodres.2009.10.013.

[fsn371565-bib-0057] Mulder, R. 2019. “Consumer Perceptions and Demand for Locally‐Grown Vegetables in South Dakota.” Electronic Theses and Dissertations. https://openprairie.sdstate.edu/etd/3653.

[fsn371565-bib-0058] Murugesan, L. , D. Williams‐Hill , and A. Prakash . 2011. “Effect of Irradiation on Salmonella Survival and Quality of 2 Varieties of Whole Green Onions.” Journal of Food Science 76, no. 6: M439–M444. 10.1111/j.1750-3841.2011.02216.x. 21623790

[fsn371565-bib-0059] Nadkarni, P. M. , L. Ohno‐Machado , and W. W. Chapman . 2011. “Natural Language Processing: An Introduction.” Journal of the American Medical Informatics Association 18, no. 5: 544–551. 10.1136/amiajnl-2011-000464. 21846786 PMC3168328

[fsn371565-bib-0060] Nicola, S. , and E. Fontana . 2010. “Global Horticulture: Challenges and Opportunities.” Acta Horticulturae 856: 49–54. 10.17660/ActaHortic.2010.856.5.

[fsn371565-bib-0061] Ospina‐Sanchez, P. A. , J. C. Henao‐Rojas , and J. G. Ramírez‐Gil . 2025. “Approach to the Concept of Multidimensional Quality in Carrots Through Digital Tools With a Geospatial Component.” Food Science & Nutrition 13, no. 8: e70718. 10.1002/fsn3.70718. 40741091 PMC12307244

[fsn371565-bib-0062] Panthee, D. R. , J. A. Labate , M. T. McGrath , A. P. Breksa , and L. D. Robertson . 2013. “Genotype and Environmental Interaction for Fruit Quality Traits in Vintage Tomato Varieties.” Euphytica 193, no. 2: 169–182. 10.1007/s10681-013-0895-1.

[fsn371565-bib-0063] Pereira, V. , M. P. Basilio , and C. H. T. Santos . 2023. “pyBibX—A Python Library for Bibliometric and Scientometric Analysis Powered With Artificial Intelligence Tools.” Data Technologies and Applications. 10.1108/DTA-08-2023-0461.

[fsn371565-bib-0065] Putnik, P. , D. Gabrić , S. Roohinejad , et al. 2019. “An Overview of Organosulfur Compounds From *Allium* spp.: From Processing and Preservation to Evaluation of Their Bioavailability, Antimicrobial, and Anti‐Inflammatory Properties.” Food Chemistry 276: 680–691. 10.1016/j.foodchem.2018.10.068. 30409648

[fsn371565-bib-0066] Raman, G. , E. E. Avendano , S. Chen , et al. 2019. “Dietary Intakes of Flavan‐3‐Ols and Cardiometabolic Health: Systematic Review and Meta‐Analysis of Randomized Trials and Prospective Cohort Studies.” American Journal of Clinical Nutrition 110, no. 5: 1067–1078. 10.1093/ajcn/nqz178. 31504087 PMC6821550

[fsn371565-bib-0067] Ramírez‐Gil, J. G. , G. Franco , and J. C. Henao‐Rojas . 2019. “Review of the Concept of Quality in Hass Avocado and the Pre‐Harvest and Harvest Factors That Determine It Under Tropical Conditions.” Revista Colombiana de Ciencias Hortícolas 13, no. 3: 359–370. 10.17584/rcch.2019v13i3.10503.

[fsn371565-bib-0068] Reynolds, W. 2023. “Emerging IoT Technologies for Smart Agriculture–Delaware State University.” Annual Reporting Frequency. National Institute of Food and Agriculture. https://portal.nifa.usda.gov/web/crisprojectpages/1028602‐emerging‐iot‐technologies‐for‐smart‐agriculture.html.

[fsn371565-bib-0069] Rocchetti, G. , L. Zhang , S. Bocchi , et al. 2022. “The Functional Potential of Nine *Allium* Species Related to Their Untargeted Phytochemical Characterization, Antioxidant Capacity and Enzyme Inhibitory Ability.” Food Chemistry 368: 130782. 10.1016/j.foodchem.2021.130782. 34392121

[fsn371565-bib-0070] Sánchez, J. , A. Acuña , and J. Hernández . 2019. “Poscosecha en colombia: Tendencias y expansión global.” https://repositorio.sena.edu.co/handle/11404/8300.

[fsn371565-bib-0071] Schaub, S. , R. Huber , and R. Finger . 2020. “Tracking Societal Concerns on Pesticides – A Google Trends Analysis.” Environmental Research Letters 15, no. 8: 84049. 10.1088/1748-9326/ab9af5.

[fsn371565-bib-0072] Segura, M. , J. C. Lesmes , J. R. Galindo , et al. 2015. Modelo tecnológico para el cultivo de cebolla de rama (Allium fistulosum L.) en el departamento de Boyacá. Corporación colombiana de investigación agropecuaria ‐ AGROSAVIA. https://repository.agrosavia.co/handle/20.500.12324/13763.

[fsn371565-bib-0073] Skarlinski, M. , T. Nadolski , J. Braza , et al. 2025. “FutureHouse Platform: Superintelligent AI Agents for Scientific Discovery.” https://www.futurehouse.org/research‐announcements/launching‐futurehouse‐platform‐ai‐agents?utm_source=chatgpt.com.

[fsn371565-bib-0074] Steinke, J. , J. van Etten , and P. M. Zelan . 2017. “The Accuracy of Farmer‐Generated Data in an Agricultural Citizen Science Methodology.” Agronomy for Sustainable Development 37, no. 4: 32. 10.1007/s13593-017-0441-y.

[fsn371565-bib-0075] Thirunavukarasu, A. J. 2021. “Evaluating the Mainstream Impact of Ophthalmological Research With Google Trends.” Eye 35, no. 11: 3165–3167. 10.1038/s41433-020-01257-4. 33132384 PMC7603637

[fsn371565-bib-0093] Toivonen, P. M. A. , and D. A. Brummell . 2008. “Biochemical Bases of Appearance and Texture Changes in Fresh‐Cut Fruit and Vegetables.” Postharvest Biology and Technology 48, no. 1: 1–14. 10.1016/j.postharvbio.2007.09.004.

[fsn371565-bib-0076] University of California . 2025. Onions, Green Bunch. Postharvest Research and Extension Center. https://postharvest.ucdavis.edu/ar/produce‐facts‐sheets/onions‐green‐bunch?utm_source=chatgpt.com.

[fsn371565-bib-0077] Vahdanjoo, M. , C. G. Sørensen , and M. Nørremark . 2025. “Digital Transformation of the Agri‐Food System.” Current Opinion in Food Science 63: 101287. 10.1016/j.cofs.2025.101287.

[fsn371565-bib-0078] Vazquez‐Prieto, M. A. , and R. M. Miatello . 2010. “Organosulfur Compounds and Cardiovascular Disease.” Molecular Aspects of Medicine 31, no. 6: 540–545. 10.1016/j.mam.2010.09.009. 20940019

[fsn371565-bib-0079] Venu, S. , S. Khushbu , S. Santhi , A. Rawson , C. K. Sunil , and K. Sureshkumar . 2019. “Phytochemical Profile and Therapeutic Properties of Leafy Vegetables.” In Plant and Human Health, Volume 2: Phytochemistry and Molecular Aspects, edited by M. Ozturk and K. R. Hakeem , 627–660. Springer International Publishing. 10.1007/978-3-030-03344-6_26.

[fsn371565-bib-0081] Wang, H. , Q. Zheng , A. Dong , J. Wang , and J. Si . 2023. “Chemical Constituents, Biological Activities, and Proposed Biosynthetic Pathways of Steroidal Saponins From Healthy Nutritious Vegetable—Allium.” Nutrients 15, no. 9: 9. 10.3390/nu15092233. PMC10180908 37432450

[fsn371565-bib-0082] Wang, J. , L. Qiao , B. Liu , et al. 2023. “Characteristic Aroma‐Active Components of Fried Green Onion (*Allium fistulosum* L.) Through Flavoromics Analysis.” Food Chemistry 429: 136909. 10.1016/j.foodchem.2023.136909. 37516048

[fsn371565-bib-0083] Wong, D. 2018. “VOSviewer.” Technical Services Quarterly 35, no. 2: 219–220. 10.1080/07317131.2018.1425352.

[fsn371565-bib-0084] Xi, X. , S. Hu , X. Zhang , et al. 2025. “Bioactives in Food‐As‐Medicine for Special Medical Purposes.” Advances in Nutrition 16, no. 12: 100546. 10.1016/j.advnut.2025.100546. 41101394 PMC12766056

[fsn371565-bib-0085] Yaguchi, S. , T. T. M. Hang , H. Tsukazaki , et al. 2009. “Molecular and Biochemical Identification of Alien Chromosome Additions in Shallot (*Allium cepa* L. Aggregatum Group) Carrying Extra Chromosome(s) of Bunching Onion (*A. fistulosum* L.).” Genes & Genetic Systems 84, no. 1: 43–55. 10.1266/ggs.84.43. 19420800

[fsn371565-bib-0086] Yan, J.‐K. , J. Zhu , Y. Liu , et al. 2023. “Recent Advances in Research on Allium Plants: Functional Ingredients, Physiological Activities, and Applications in Agricultural and Food Sciences.” Critical Reviews in Food Science and Nutrition 63, no. 26: 8107–8135. 10.1080/10408398.2022.2056132. 35343832

[fsn371565-bib-0087] Yang, B. , and Y. Xu . 2021. “Applications of Deep‐Learning Approaches in Horticultural Research: A Review.” Horticulture Research 8: 123. 10.1038/s41438-021-00560-9. 34059657 PMC8167084

[fsn371565-bib-0096] Yang, M. H. , N.‐H. Kim , J.‐D. Heo , et al. 2017. “Comparative Evaluation of Sulfur Compounds Contents and Antiobesity Properties of Allium hookeri Prepared by Different Drying Methods.” Evidence‐based Complementary and Alternative Medicine: Ecam 2017: 2436927. 10.1155/2017/2436927. 28400840 PMC5376446

[fsn371565-bib-0088] Zhao, C. , Z. Wang , R. Cui , et al. 2021. “Effects of Nitrogen Application on Phytochemical Component Levels and Anticancer and Antioxidant Activities of *Allium fistulosum* .” PeerJ 9: e11706. 10.7717/peerj.11706. 34221743 PMC8236235

[fsn371565-bib-0089] Zhao, X. , J. Peng , L. Zhang , et al. 2024. “Optimizing the Quality of Horticultural Crop: Insights Into Pre‐Harvest Practices in Controlled Environment Agriculture.” Frontiers in Plant Science 15: 1427471. 10.3389/fpls.2024.1427471. 39109059 PMC11300219

