# Supplementary material for: Multidimensional Perspective on the Quality of Green Onion: Between the Visible, the Measurable, and the Perceived
Source: Food Sci Nutr. 2026 Feb 19;14(2):e71565. doi: 10.1002/fsn3.71565 (PMC12920262; doi:10.1002/fsn3.71565)
Supplement: Supplementary file 1 — Figure S1: Thematic and geographic analysis of scientific production on Allium fistulosum L. (2001–2025). (A) Evolution of research topics related to green onion quality. (B) Distribution of most productive authors by country. (C) Timeline of scientific productivity per author. [file FSN3-14-e71565-s001.docx]

**Supplementary material**


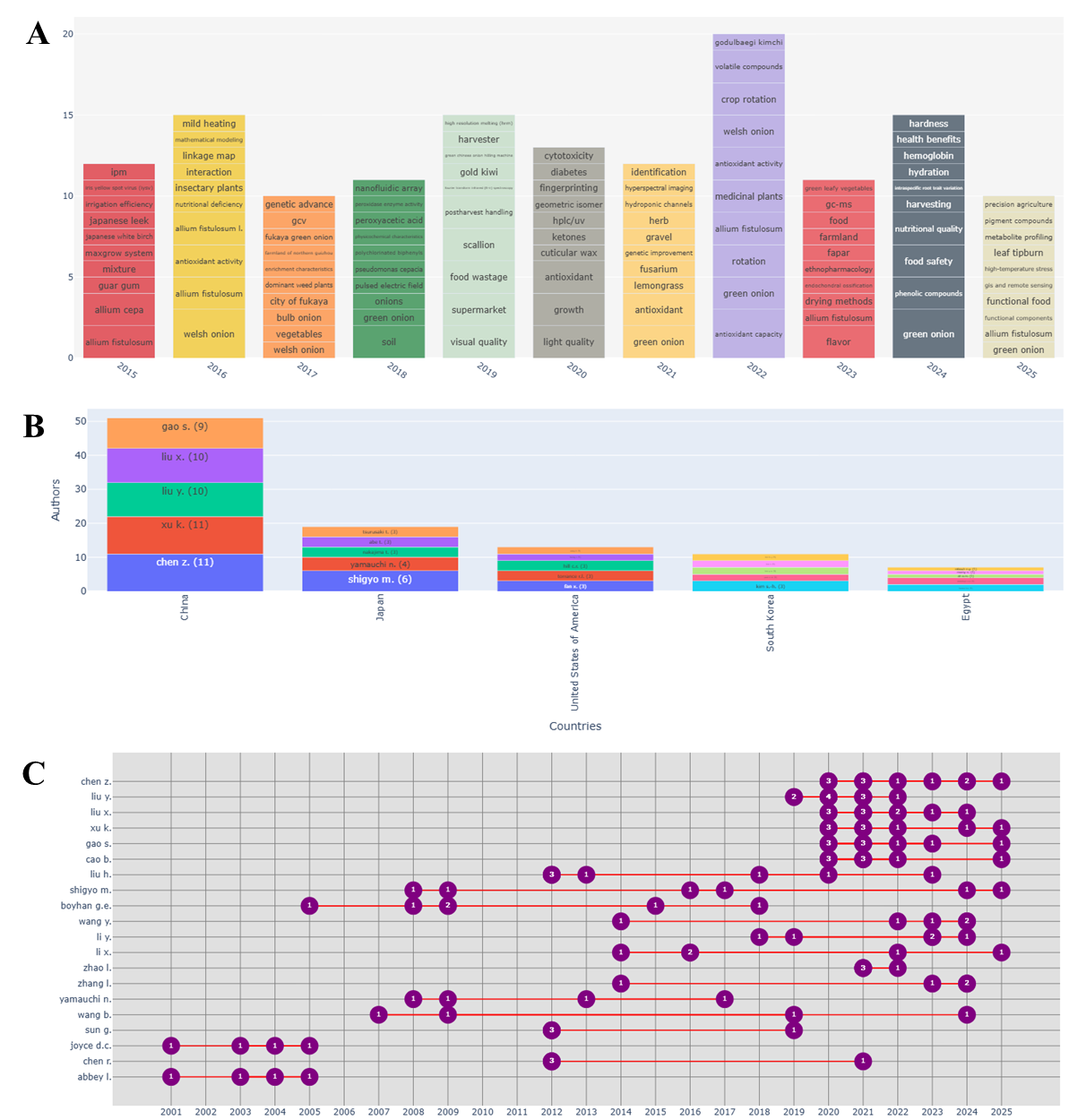


**Supplementary Figure 1.** Thematic and geographic analysis of scientific production on Allium fistulosum L. (2001–2025). (A) Evolution of research topics related to green onion quality. (B) Distribution of most productive authors by country. (C) Timeline of scientific productivity per author.
